# Natural Killer Cell Lytic Granule Secretion Occurs through a Pervasive Actin Network at the Immune Synapse

**DOI:** 10.1371/journal.pbio.1001151

**Published:** 2011-09-13

**Authors:** Gregory D. Rak, Emily M. Mace, Pinaki P. Banerjee, Tatyana Svitkina, Jordan S. Orange

**Affiliations:** 1 University of Pennsylvania School of Medicine, Philadelphia, Pennsylvania, United States of America; 2 Children's Hospital of Philadelphia Research Institute, Children's Hospital of Philadelphia, Philadelphia, Pennsylvania, United States of America; 3 Department of Biology, University of Pennsylvania, Philadelphia, Pennsylvania, United States of America; National Jewish Medical and Research Center/Howard Hughes Medical Institute, United States of America

## Abstract

Accumulation of filamentous actin (F-actin) at the immunological synapse (IS) is a prerequisite for the cytotoxic function of natural killer (NK) cells. Subsequent to reorganization of the actin network, lytic granules polarize to the IS where their contents are secreted directly toward a target cell, providing critical access to host defense. There has been limited investigation into the relationship between the actin network and degranulation. Thus, we have evaluated the actin network and secretion using microscopy techniques that provide unprecedented resolution and/or functional insight. We show that the actin network extends throughout the IS and that degranulation occurs in areas where there is actin, albeit in sub-micron relatively hypodense regions. Therefore we propose that granules reach the plasma membrane in clearances in the network that are appropriately sized to minimally accommodate a granule and allow it to interact with the filaments. Our data support a model whereby lytic granules and the actin network are intimately associated during the secretion process and broadly suggest a mechanism for the secretion of large organelles in the context of a cortical actin barrier.

## Abstract 2

Super-resolution imaging provides a new look at how the lytic granules in natural killer cells penetrate the filamentous actin network of the immunological synapse.

## Author Summary

The immune system's natural killer cells eliminate diseased cells in the body. They do so by secreting toxic molecules directly towards the diseased cells, so causing their death. This process is essential for the host organism to defend itself against infectious diseases. The interface between the natural killer cell and its target—the lytic immunological synapse—forms by close apposition of the surface membranes of the two cells. It is characterized by coordinated rearrangement of proteins to allow lytic granules, which contain the toxic molecules, to fuse with the cell surface at the synapse. Given the large size of the granules, one challenge the natural killer cell faces is how to contend with network of actin filaments just under the cell surface, which potentially could pose a barrier to secretion. The current model proposes large-scale clearing of actin filaments from the center of the immunological synapse to provide granules access to the synaptic membrane. By using very high-resolution imaging techniques, we now demonstrate that actin filaments are present throughout the synapse and that natural killer cells overcome the actin barrier not by wholesale clearing but by making minimally sufficient conduits in the actin network. This suggests a model in which granules access the surface membrane by means of specific and facilitated contact with the actin cytoskeleton.

## Introduction

Natural killer (NK) cells are lymphocytes of the innate immune system that function in clearance of tumor and virally infected cells [1]. Elimination of susceptible target cells is tightly regulated and follows ligation of germline-encoded activation receptors [2]. As NK cells do not require receptor gene rearrangement, they are constitutively enabled for cytotoxicity. Thus, NK cell activation must be tightly regulated to ensure that healthy cells remain unharmed. Efficient lysis requires the tight adherent formation between the NK cell and the target cell termed the immunologic synapse (IS). The formation of a mature, cytolytic synapse between an NK cell and a target cell occurs in stages that can be thought of as checkpoints in the activation process [3]–[5]. Major cytoskeletal steps that are required in this process include the rearrangement of filamentous actin (F-actin) and the polarization of the microtubule organizing center (MTOC) [6]–. These events culminate in the directed secretion of lytic granule contents at the IS, which is prerequisite for NK cell cytotoxicity.

F-actin accumulation at the synapse is the first major cytoskeletal reorganization event and is critical to subsequent steps and function of the IS [5]. Inhibiting proper F-actin dynamics in NK cells with the actin targeting drugs cytochalasin [6],[9], latrunculin [10], or jasplakinolide [3] inhibits their cytotoxicity. Furthermore, NK cells from patients with Wiskott-Aldrich Syndrome (WAS) who have mutations in the actin regulatory protein, WAS protein (WASp), are poorly cytotoxic [9]. This defect is attributable to improper reorganization of F-actin at the IS. Additionally, the actin nucleator Arp2/3 complex, which is enabled by WASp, is also required for cytotoxicity [10]. Cytochalasin treatment, Arp2/3 complex depletion, or WASp deficiency prevent the normal accumulation of F-actin at the synapse [5],[9],[10].

One question that arises from the creation of a dense polarized network at the IS is how secretion of lytic granules occurs through a potential barrier. The traditional view of granule delivery through the actin network holds that granules reach the synaptic membrane through a void of actin in the center of the network. This model is based on the observation from 3-D confocal microscopy that actin forms a dense peripheral ring around the IS [5],[11]. There is a caveat to the seemingly unobstructed access to the membrane that this “ring” provides: the actin motor protein, myosin IIA, is required for secretion and, more specifically, for granule delivery to the synaptic plasma membrane [12],[13]. These data are at apparent odds with one another as a requirement for myosin IIA for secretion necessitates a requirement for actin. One explanation is that granules are secreted at the periphery of the synapse where the traditional model depicts the location of F-actin. Another explanation is that the center of the synapse actually contains F-actin but does so at a level that has been undetectable by conventional 3-D confocal microscopy.

Here we use microscopy techniques that provide enhanced sensitivity and resolution over those used previously to investigate the NK cell IS. We show that F-actin is present throughout the synapse and that lytic granules likely navigate and are secreted through the filamentous network by accessing minimally sufficiently sized clearances. These data demonstrate a previously unappreciated distribution of F-actin at the NK cell IS and redefine granule access to the synaptic membrane and functional secretion.

## Results

### Actin Accumulates throughout the Activated Immunological Synapse

**Figure 1:**
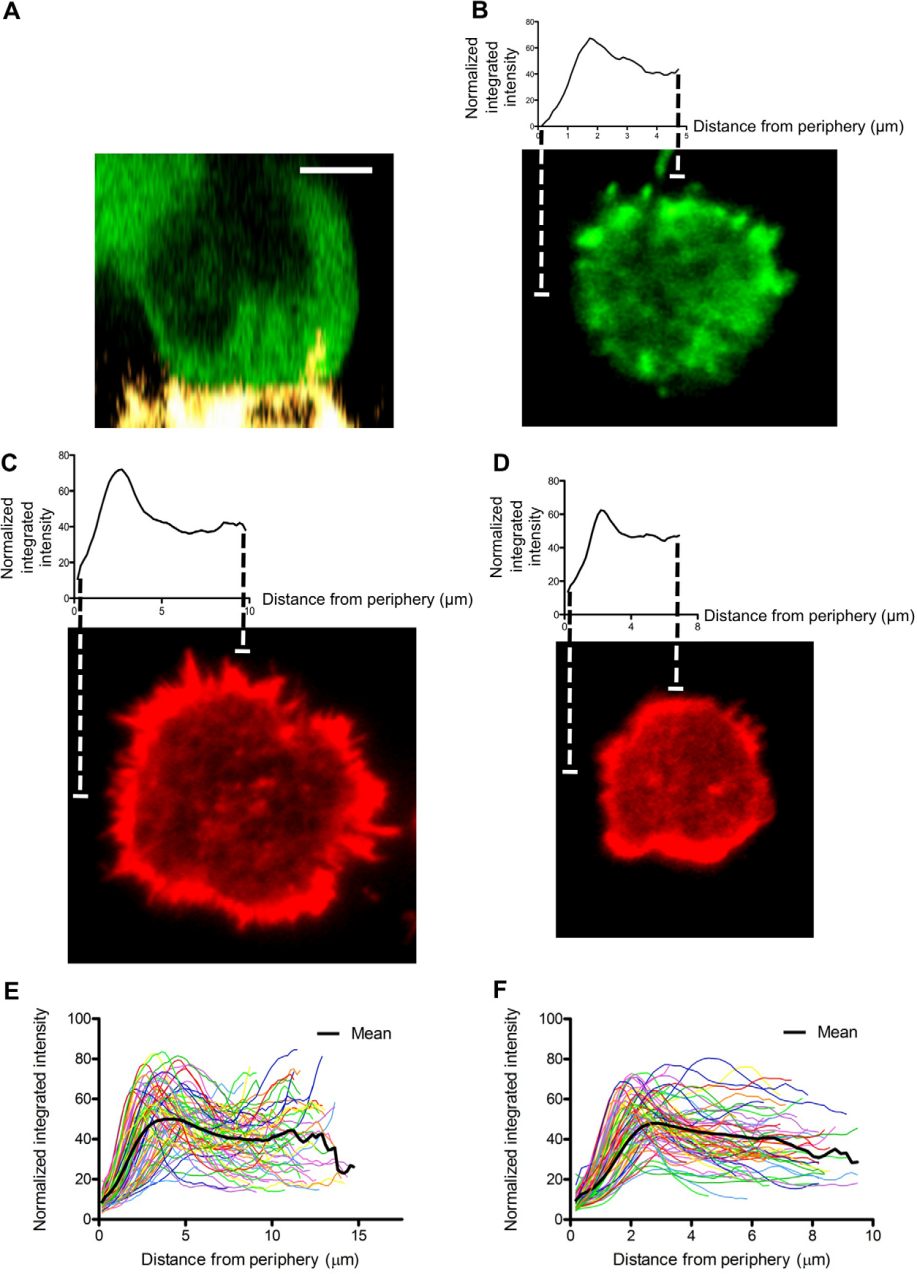
Actin distribution at the activated NK cell IS. (A) 3-D projection of NK-92 GFP-actin expressing cell (green) conjugated to mel1190 target cell (yellow). Scale bar = 5 m. (B) X–Y projection of a synapse taken from a conjugate similar to (A). (C,D) X–Y projections of a representative NK-92 (left) or ex vivo (right) cell that had been activated for 30 min at 37°C on immobilized antibody to NKp30 and CD18, fixed, stained with 568 phalloidin (red), and imaged by TIRF microscopy. Above each X–Y projection is a plot of the mean fluorescent intensity (MFI) of concentric circles at a given distance beginning at the periphery of the cell and moving inward to the center (radial intensity profiles). (E,F) Plot of radial intensity profiles from 70 cells from 3 experiments for each cell type. Mean value is shown in black.

Visualization of the synaptic actin network has relied on 3-D reconstructions of confocal slices [5],[11]. Here, we took advantage of the superior resolution of imaging in the XY plane to investigate the polarization and distribution of actin. First, we evaluated GFP-actin expressing NK-92 cells conjugating with the susceptible and adherent cell line, mel1190. These cells conjugated in a manner that afforded us the ability to image the synapse in the XY plane. GFP-actin was polarized toward the contact site (Figure 1A) and surprisingly displayed a diffuse distribution across the synapse (Figure 1B). This distribution was quantitatively analyzed and confirmed using a radial intensity profiling algorithm, which demonstrated that the intensity throughout the contact site was substantially above the background.

To more directly image the cortical region of the NK cell immunologic synapse, we used total internal reflection fluorescence microscopy (TIRFm), which has the benefit of an improved signal to noise ratio over confocal microscopy and is limited to visualization within the first membrane proximal 100 nm [14]. Cells were activated via crosslinking of NKp30, a natural cytotoxicity receptor whose ligand is expressed on tumor cells [15], and CD18, a member of the heterodimeric integrin lymphocyte function-associated antigen-1 (LFA-1). Both integrin receptor and activation receptor activation are critical for polarized secretion of granule contents [16]. This combination of signals resulted in robust activation, which was demonstrated by degranulation as measured by enzymatic activity of granzyme A in the supernatant (Figure S1). TIRFm imaging of activated NK-92 cells demonstrated a distribution of F-actin throughout the synapse (Figure 1C). Quantitative analysis using radial profile plotting confirmed the presence of F-actin throughout the cell contacts (Figure 1E). To ensure that these findings were not particular to the NK-92 cell line, we activated and imaged freshly isolated ex vivo NK cells. Similar to NK-92 cells, synaptic F-actin in ex vivo NK cells was identified throughout the contact (Figure 1D,F). These results demonstrate that the NK cell synapse is defined by an abundant, diffuse F-actin network.

To evaluate the kinetics of actin accumulation at the activated synapse, NK-92 cells expressing GFP-actin were imaged using TIRFm after contacting an activating surface. Actin accumulated quickly, within 5 min, and was sustained over the period of observation (50 min) (Figure S2A, Video S1). There was an initial paucity of actin at the synapse followed by a rapid filling in, as demonstrated by the separation of peak contact area and mean fluorescence intensity (MFI) of GFP-actin in that region (Figure S2B). The decrease in MFI over time was due to photobleaching as separate imaging of fields at 10 and 40 min did not show MFI differences (unpublished data). Importantly, actin was diffusely accumulated prior to timepoints at which granule contents were detected in the supernatant (Figure S1). Thus, actin was present as a potential barrier to lytic granule access to the plasma membrane.

### Lytic Granules Approximate the Synapse in Areas of Actin

**Figure 2:**
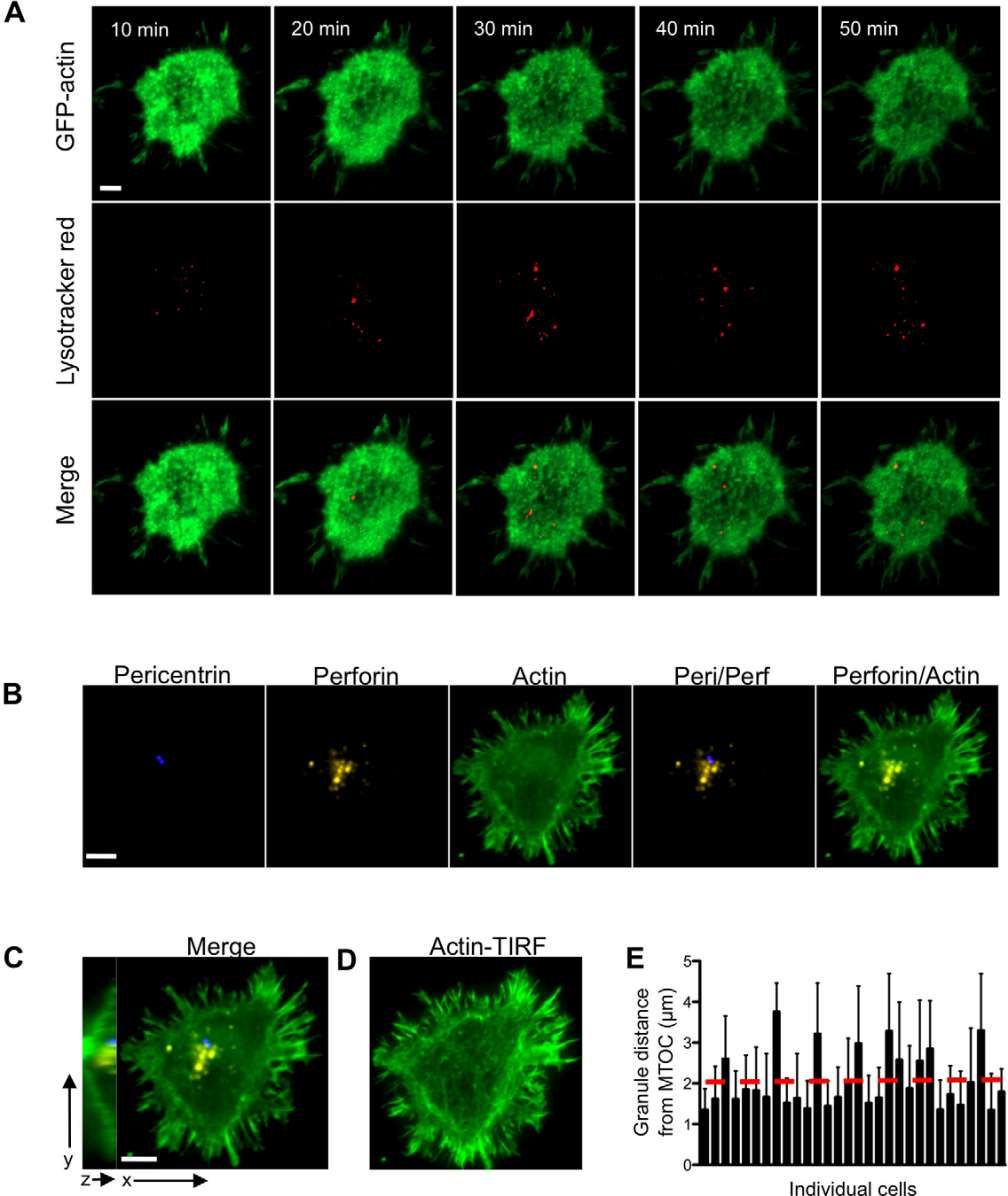
Granule approach to the synaptic membrane is coincident with the MTOC and actin network. (A) NK-92 cells expressing GFP-actin (green) were loaded with LysoTracker Red (red) and activated by immobilized antibody to NKp30 and CD18. Cells were imaged at 1 frame per minute for 60 min using TIRFm. Images are shown at 10 min intervals starting at 10 min following contact. Images for the GFP channel (top), LysoTracker Red channel (middle), and a merge of both (bottom) are shown. (B–E) NK-92 cells were activated on glass, fixed, stained, and imaged by both confocal and TIRF microscopy using a 100× objective. (B) Images show pericentrin (blue), perforin (yellow), actin (green), and indicated merges at the plane of contact with the glass. (C) Full merge from (B) is shown to the right of a Y–Z projection of the cell. (D) TIRFm image of the cell from (B, C). (E) Distances of granules to the MTOC were measured and averaged on a per cell basis for 30 cells over 2 experiments. The red dashed line denotes the mean of all cells. Scale bars = 5 µm.

Because there was abundant actin present at the synapse, we wanted to determine if lytic granules might utilize relative clearances in the actin network to access the synaptic membrane. To address this, GFP-actin expressing cells were loaded with LysoTracker Red dye, which enables tracking of lytic granules and definition of their position relative to actin, and followed in real time after activation. Numerous granules were identified in the synaptic actin network using two-color TIRFm. Although some relative hypodensities were apparent in the synaptic actin network (Figure S3A–C), the LysoTracker labeled granules did not necessarily appear in these relative voids of actin (Figure 2A, Video S2). To quantitatively analyze this observation across all synaptic granules in an NK cell, the actin intensity in the region of the synaptic granule was compared to that of the entire synapse by dividing the MFI of the respective intensity values to produce a ratio measurement. This ratio, when compared to minimum and maximum potential ratios, demonstrated that on average granules approached the membrane in areas of actin (Figure S4A,B). Combining measurements of all granules in the synapse over 1 h from 14 cells defined the mean granule ratio value as 1.0 (Figure S4C). Although there was a range of actin intensities present throughout the synapse as measured by the ratios of minimum and maximum intensity values to the MFI, few granules were present in areas of particularly low or high actin content. Thus, the colocalization of lytic granules with mean actin signal suggested that granules access the synapse in close proximity to the actin network.

### The MTOC Delivers Granules to the Synaptic Actin Network

The MTOC is known to deliver lytic granules to the immunological synapse in NK cells [17]. To investigate the relationship among granules, the MTOC, and the synaptic actin network, we imaged the synapse using both confocal microscopy and TIRFm. The MTOC was present in the plane of the synapse and granules that were also in the plane of the synapse were present at a mean distance of 2.05 µm from the MTOC (Figure 2B,C,E), a distance consistent with granules that are converged to the MTOC [17]. To note, the MTOC was not present in any distinct clearance of F-actin. TIRFm demonstrated more clearly than confocal microscopy the varying density of the synaptic actin network (Figure 2D). These data suggest that the MTOC delivers granules to the synaptic actin network.

### pHluorin-LAMP1 as an Indicator of Degranulation

Because there was variability in colocalization between synaptic actin and granules (Figure S4), we considered the possibility that an approximated granule might not necessarily be capable of degranulation. Specifically, we reasoned that granules that ultimately degranulate represent a subpopulation of approximation events. Furthermore, we hypothesized that granules capable of degranulation might be those present within focal actin hypodensities. In order to study this directly, we developed a novel degranulation indicator for use in live cells.

**Figure 3:**
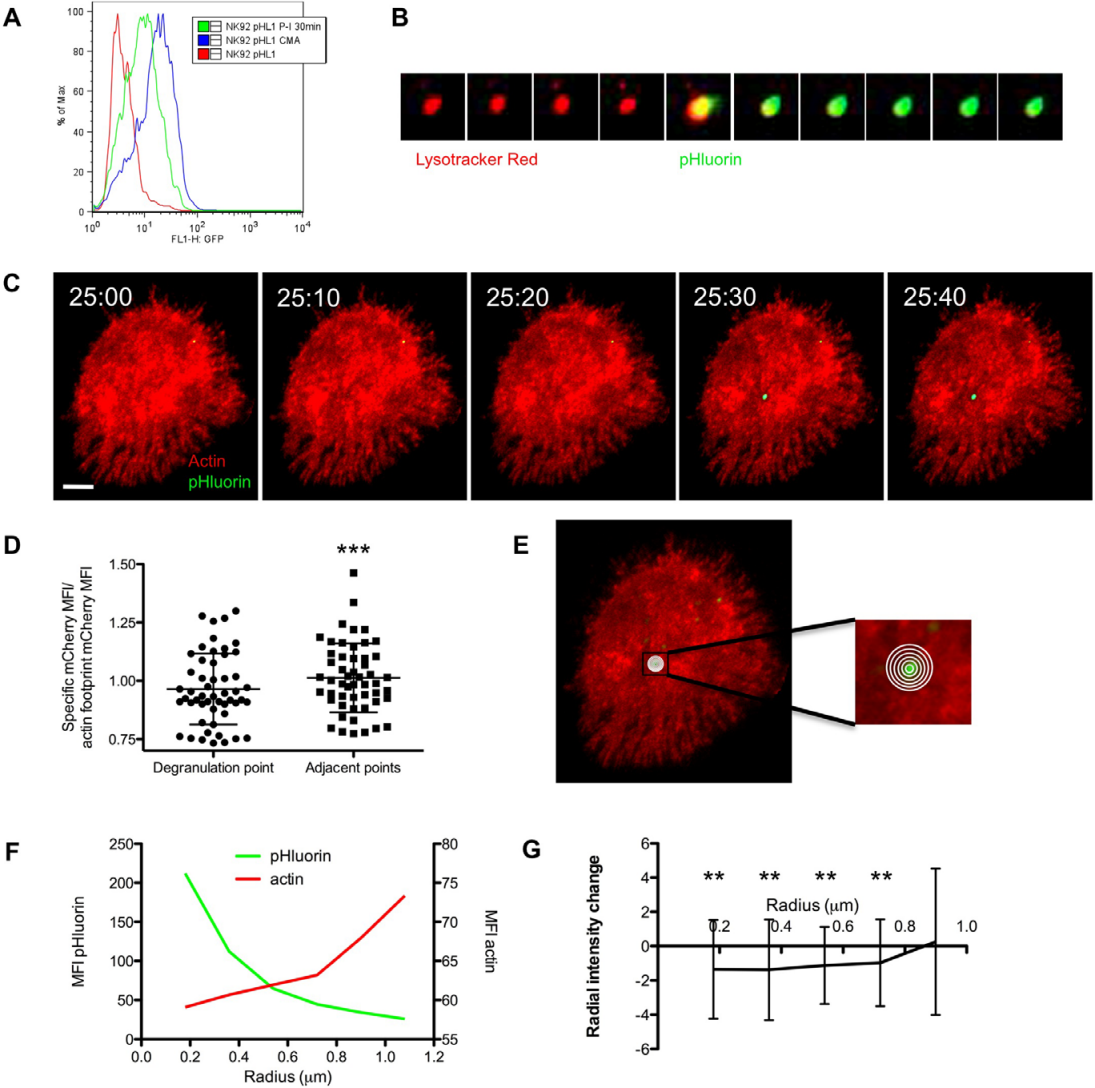
pHluorin-LAMP1 reports degranulation in locally hypodense regions of actin. (A) Histogram demonstrating green fluorescence measured by flow cytometry of NK-92 cells expressing pHluorin-LAMP1. Cells were untreated or treated with PMA/Ionomycin or CMA. (B) NK-92 cells expressing pHluorin-LAMP1 (green) were loaded with LysoTracker Red (red) and imaged by TIRF under activating conditions. Frames were acquired at a rate of 2 frames per min following 10 min of activation and the image sequence depicts a cropped section showing a single lytic granule over time. (C) NK-92 cells expressing pHluorin-LAMP1 and mCherry-actin were activated by immobilized antibody to NKp30 and CD18 and imaged by TIRFm. Images shown were taken at 10-s intervals at indicated time of activation. Scale bar = 5 µm. (D) Ratio measurements of the MFI of mCherry-actin at the site of degranulation, or adjacent points, to the MFI of the whole cell footprint were calculated and represent 52 events; means = 0.965 and 1.013, respectively. (*** *p* = 0.0001, paired *t* test). (E) Image from (A) overlaid with concentric circles starting from the centroid of a degranulation event demonstrates measurement strategy for regional actin fluorescence intensity. (F) Radial intensity profiles of pHluorin and mCherry MFI are depicted and show the signal intensity changes as circles are moved radially outward from the centroid of degranulation event (left to right). (G) Radial intensity change of sequential circles moving outward from the centroid. Data represent 52 degranulation events (error bars, ± SD). Values are statistically significantly (** *p*<0.01, one sample *t* test) different to a value of 0.0, which would represent no change between sequential circles.

Lysosomal-associated membrane protein 1 (LAMP1, CD107a), which is sorted to lytic granules [18], is routinely used to detect cells that have degranulated by its appearance on the cell surface [19],[20]. Although previous investigations used antibody to LAMP1 to visualize degranulation [21], we adopted a cell-intrinsic approach by targeting a reporter fluorophore to the lytic granules. We fused pHluorin, a pH sensitive mutant of GFP that does not fluoresce at acidic pH [22], to the cytoplasmic tail of LAMP1 (Figure S5A,B) and obtained stable expression in NK-92 cells. As expected with localization of the pHluorin-LAMP1 construct to lytic granules, treatment with concanamycin A (which effectively neutralizes lysosomal pH by inhibiting the vacuolar-type H+ ATPase [23]) resulted in a robust increase in green fluorescence as measured by flow cytometry (Figure 3A). Since degranulation is an activation-induced process, we also treated pHluorin-LAMP1 expressing cells with the phorbol ester, PMA, and calcium ionophore, ionomycin, and found a rapid increase in pHluorin fluorescence, consistent with LAMP1 surface upregulation (Figure 3A). To better define pHluorin-LAMP1 localization to acidic granules, LysoTracker Red loaded pHluorin-LAMP1 expressing cells were studied using TIRFm after activation. Individual LysoTracker Red labeled granules could be identified at the synapse and were observed to undergo a shift from red to green fluorescence (Figure 3B, Video S3). This event is consistent with the granule fusing with the synaptic membrane, releasing its contents, and encountering a pH neutral environment. These data are consistent with lytic granule targeting of pHluorin.

We next used pHluorin-LAMP1 expressing NK-92 cells to address whether granule approximation results in degranulation. LysoTracker Red loaded, pHluorin-LAMP1 expressing cells were imaged over time using TIRFm (Figure S6A, Video S4). There were significantly more approximation events than degranulation events (mean = 31 and 8 per cell, respectively) over 1 h (Figure S6B,C). Thus only a subset of granules that approximate the synaptic membrane result in a degranulation event.

### Degranulation Occurs within Focal Actin Network Hypodensities

To directly investigate where degranulation occurs relative to the synaptic actin network, we stably coexpressed pHluorin-LAMP1 and mCherry-actin in cells and imaged them following activation using two-color TIRFm. Timelapse imaging demonstrated that degranulation events occurred in areas of at least some actin fluorescence, similar to that which was seen with granule approximations (Figure 3C, Video S5). We quantitatively evaluated the actin intensity at degranulation points by dividing the intensity values of the actin signal at the point of degranulation events by that of the entire cell contact (ratio of MFIs), thus generating a normalized, comparable value. Degranulations were identified in regions of actin that had slightly lower signal than the mean actin signal of the cell footprint (ratio = 0.965) (Figures 3D, S7). To provide an additional measure, the ratio of MFIs for points adjacent to the degranulation and the entire cell contact were calculated. When compared to ratio of MFIs for the degranulation point itself, the ratio of MFIs for adjacent points were significantly higher. This indicated that degranulation events were occurring in areas of locally hypodense actin. We additionally calculated the minimum and maximum potential values of the ratio measurement. The degranulation values were between the minimum and maximum values (Figure S7). This indicated that degranulation events did not occur in areas of minimal nor maximal actin but rather that they occurred in close proximity to the actin network.

To further characterize the local actin network at the point of degranulation in consideration of focal hypodense regions, we quantitatively evaluated actin fluorescence in the entire immediate vicinity of degranulation events. Measurements of actin fluorescence were made along sequential pixel radii emanating from the centroid of individual degranulations extending approximately 1 µm outwards (Figure 3E). The fluorescent intensities of actin as well as that of pHluorin were quantified in concentric circles along these radii (Figure 3F). In general, as the pHluorin signal diminished from a degranulation centroid, the mCherry-actin signal increased suggesting that degranulation occurs in a focal actin hypodensity (Figure 3F). To measure multiple events, the change in intensity between consecutive radiating circles was determined and plotted for all observed degranulation events (Figure 3G). The mean value of the intensity change demonstrated reduced actin intensity at each of the innermost four radii compared to the neighboring outer radius, thus reflecting the example in Figure 3F. These values were significantly different from the baseline value of zero, which would have indicated no change in the actin network. This indicates that in moving from the periphery of the region of the degranulation event to its center the actin intensity decreased to a detectable degree. Thus, degranulation tends to occur in locally hypodense areas of the actin network (i.e., in regions with some but relatively less actin). In the presence of an actin barrier at the plasma membrane, therefore, hypodense regions of actin provide a potentially more accessible route to the synaptic membrane.

### Inhibiting Actin and Its Dynamics After Activation Inhibits Degranulation

**Figure 4:**
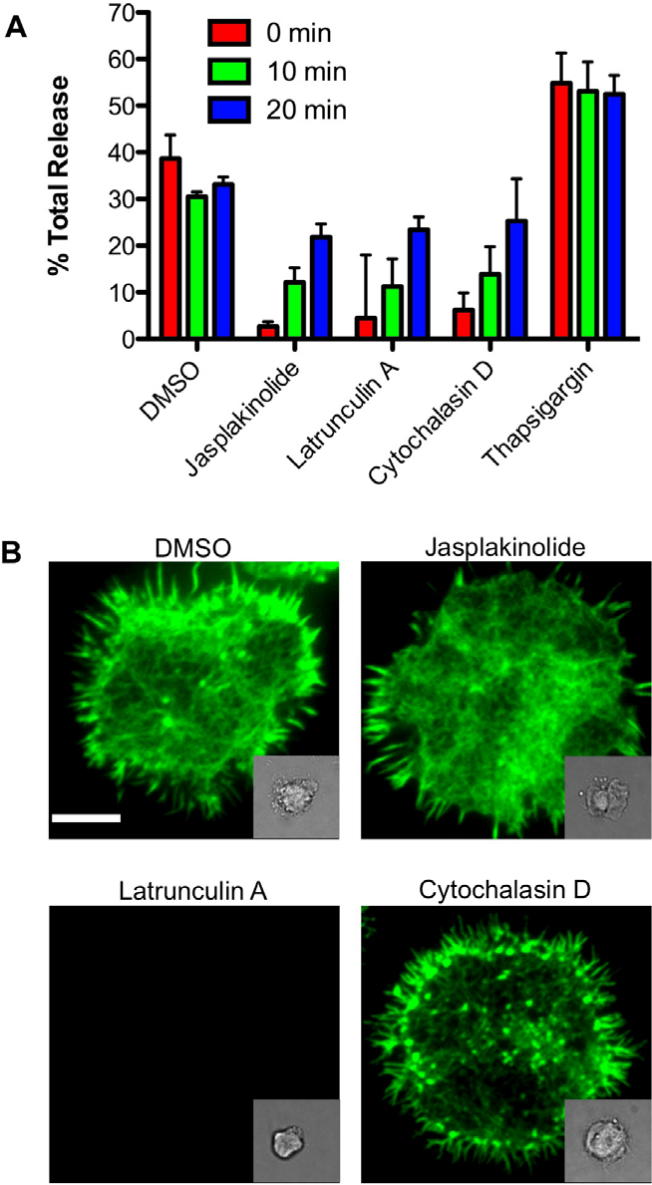
Inhibiting actin dynamics after activation interferes with degranulation. NK-92 cells were activated by immobilized antibody to NKp30 and CD18 and incubated at 37°C. (A) Indicated inhibitors were added to samples at 0 min, 10 min, or 20 min following activation. Supernatants were harvested after 60 min of total incubation and assayed for Granzyme A activity. All values are statistically significantly different from respective DMSO controls (range: *p*<0.001–0.05, unpaired *t* test) and represent the mean of three experiments (error bars = SD). (B) Activated cells were treated with DMSO or indicated inhibitor, fixed, stained for F-actin with phalloidin, and imaged by TIRFm using a 100× objective. Representative images from the 10 min timepoint are shown with respective DIC images (inset). Scale bar = 5 µm.

Since granules are in contact with at least some actin during degranulation, we next investigated the role of actin dynamics in degranulation. We inhibited actin polymerization and dynamics with drugs that prevent F-actin assembly (latrunculin A and cytochalasin D) or disassembly (jasplakinolide). Inhibitor addition at the time of activation almost completely inhibited degranulation (Figure 4A), a result that is consistent with the previously reported requirement for initial actin reorganization in synapse formation and maturation [5]. In order to avoid inhibiting the initial, requisite actin reorganization (which we observed by 5 min—Figure S2), we treated cells with the inhibitors following 10 min of activation (a point at which minimal degranulation had occurred—Figure S1). Addition of the actin inhibitors after 10 min of activation resulted in an approximately 50% decrease in bulk degranulation (Figure 4A). Interestingly, addition of inhibitors at 20 min had only a marginal effect on degranulation. Thapsigargin, which has the net effect of elevating intracellular calcium levels, was used as a positive control and increased degranulation.

To further evaluate the effects of the inhibitors on NK cells, we imaged F-actin at the synapse following inhibitor treatment. Jasplakinolide had no effect on F-actin presence or distribution; latrunculin A completely depleted the actin network; cytochalasin D had variable effects on cells, with some cells appearing unaffected while others showing a relative depletion of some filaments (Figure 4B, and unpublished data). To quantitatively evaluate these effects, cells were analyzed using radial intensity profile measurements. This analysis demonstrated no major depletion in F-actin following jasplakinolide or cytochalasin D treatment and robust depletion of the actin network following latrunculin A treatment (Figure S8A,B). Collectively, these results indicate that F-actin presence and reorganization immediately prior to the start of degranulation but after large-scale actin accumulation has occurred is critical for subsequent granule release.

### The Actin Network Is Continuously Dynamic

To determine if the synaptic actin network was dynamic at the time corresponding to degranulation, GFP-actin expressing cells were imaged by TIRFm and evaluated at both early and late timepoints of activation. Subtle but consistent changes in actin intensity were visualized over the course of imaging (Figure S9A, Video S6). Although most peaks and troughs of intensity did not appear to change over time, some shifts were detected as highlighted by surface plot rendering of intensity values (Figure S9B, Video S6). To quantify this, images from 5-min series at early and late timepoints of activation were evaluated for changing fluorescent intensities by plotting line profiles across the synapse. Temporally sequential line profiles were overlaid together on one graph, which demonstrated a constant trend in the fluorescence over time with imperfect alignment (Figure S9C,D). The variability was observed during both timeframes of activation and signified a dynamic state of actin where small changes in intensity were occurring. Variation was consistent at both 10 and 30 min after activation as defined by the standard deviation of mean intensity for each pixel across a linear profile (Figure S9E). We were able to inhibit actin dynamics to a measurable degree by using jasplakinolide to stabilize filaments and latrunculin A to prevent new filament formation (Figure S9F). Thus at the level of TIRFm, the synaptic actin network was dynamic at both early and late timepoints. This is consistent with the requirement for actin function at times after which synaptic actin accumulation has occurred.

### The Activated Immunologic Synapse Is Characterized by a Ubiquitous Filamentous Network That Contains Small Clearances

The resolution of fluorescence microscopy is diffraction limited to around 200 nm [24]. While we did detect focal hypodensities in the actin network corresponding with regions of degranulation, it was unclear if these represented true openings among the actin filaments. Thus we pursued super-resolution of the synaptic actin network to surpass the limit of diffraction. Stimulated emission depletion (STED) microscopes that utilize continuous wave (CW) fiber lasers can image with spatial resolution below 60 nm and thus provide the opportunity to investigate synaptic structures with superior resolution [25].

**Figure 5:**
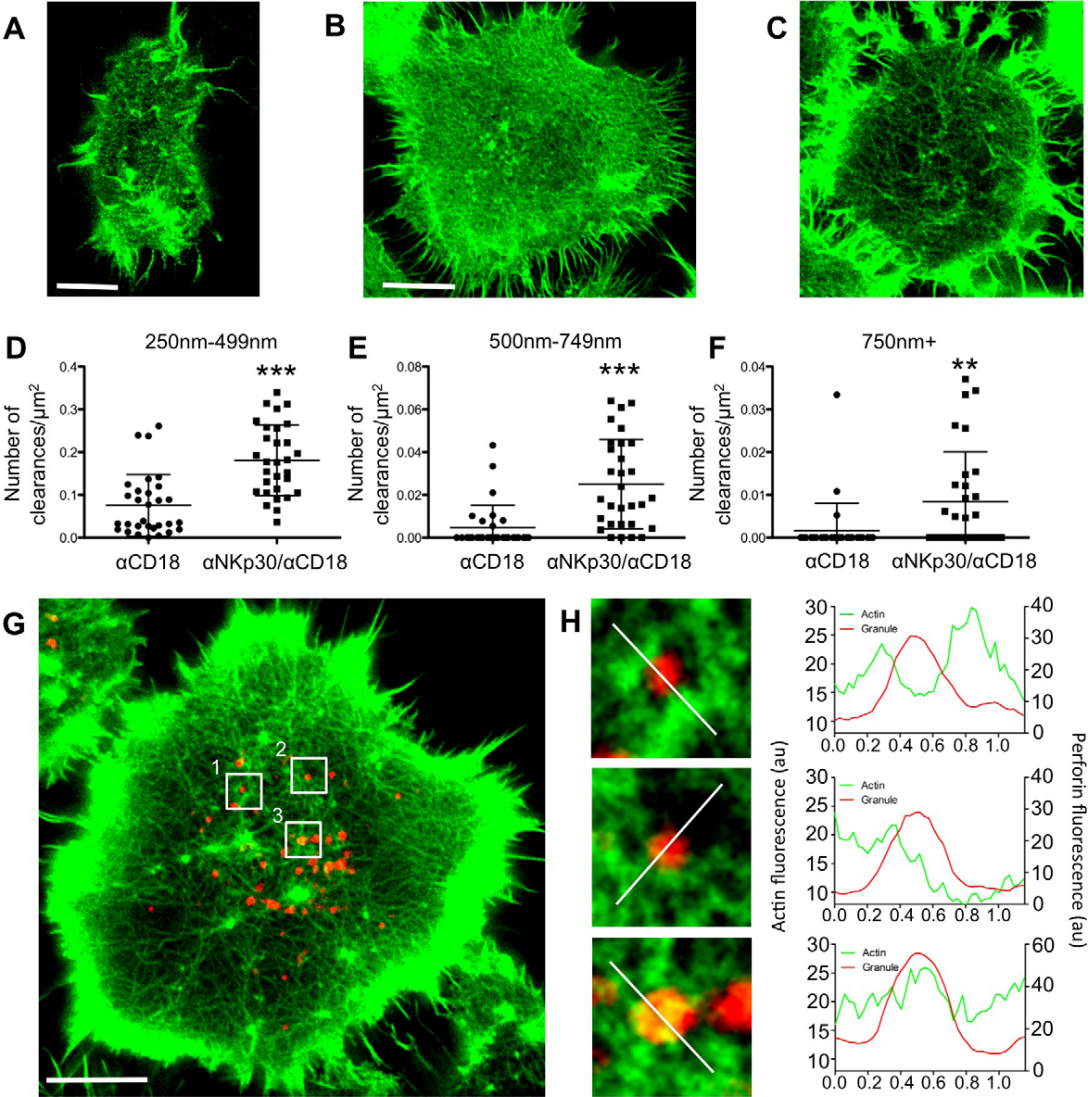
STED microscopy of the NK cell IS demonstrates activation-induced changes in the actin network and reveals varying granule locations relative to actin at the IS. (A) The synapse between a Citrine-actin expressing NK-92 and mel1190 target was imaged in the X–Y plane. (B, C) NK-92 cells were stimulated for 30 min on glass coated with either antibody to CD18 (B) or to both CD18 and NKp30 (C) and stained for F-actin with phalloidin. (D–F) Measurements of the number of clearances at the synapses of 30 cells from 2 experiments activated as in (B,C). (*** *p*<0.001; ** *p* = 0.0076; unpaired *t* test). Measurements were grouped into clearance diameters of 250–499 nm (D), 500–750 nm (E), and 750+ nm (F). (G) Activated NK-92 cell stained for F-actin (green) and perforin (red) and imaged via STED microscopy in the green channel and confocal microscopy in the red channel. (H) Magnified images are from boxes in (G): box 1 (top), box 2 (middle), and box 3 (bottom). To the right of each image is a line profile indicating pixel intensities of actin (green) and perforin (red) for the line bisecting the granules as indicated by the white line. Scale bars = 5 µm.

Using CW-STED microscopy, we first imaged Citrine-actin expressing NK-92 cells conjugated to mel1190 cells (Figure 5A). Actin was present throughout the contact and had a distribution that was similar to that seen with our confocal microscopy images (with cells activated on glass and with mel1190 cells) and TIRF images (activated on glass). This leads us to conclude that our method of activating and imaging NK cells on glass does not induce a distribution or architecture of the actin network that is distinct from that seen with actual target cells.

We next asked whether there was an activation-dependent change in the synaptic actin architecture. To this end, we imaged F-actin in cells that had been stimulated with antibody to CD18, which does not induce degranulation, or in combination with antibody to NKp30, which robustly induces degranulation (Figure S1). Cells stimulated through CD18 alone had a dense synaptic network while the addition of antibody to NKp30 resulted in a more diffuse architecture with many observable clearances in the network (Figure 5B,C). To quantify this observation, clearances were identified based on measured granule diameters. Granules were separately identified using STED microscopy and had a range of diameters with a mean value of 333±103 nm (Figure S10A). The majority of granules were in the 250–499 nm range of diameters, with a smaller number in the 500–749 nm range. These two ranges, which represent areas that are minimally sufficient in size to allow granules access to the plasma membrane, as well as the larger 750+ nm range, were used to categorize clearance area. The number of these clearances in cells significantly increased when a degranulation signal (anti-NKp30) was incorporated (Figure 5D–F). To note, there were many clearances in the 250–499 nm range, fewer in the 500–749 nm range, and still fewer to none in the large 750+ nm range upon activation (Figure 5D–F). This trend in the frequency of clearances reflects the frequency of granule diameters. Furthermore, the mean clearance size divided by the mean granule size resulted in a value of 1.48±0.48, demonstrating that on average clearances were only slightly larger than granules (Figure S10B). Thus, full activation for degranulation results in an actin network with many access points that are minimally sufficient in size to accommodate granules.

Having defined the presence of clearances in the synaptic actin network upon activation, we next sought to identify granule localization relative to the network. Thus we simultaneously imaged the actin network by STED microscopy and granules by laser scanning confocal microscopy. Granules within activated cells displayed a range of interaction with the actin network and all that were present at the synapse had at least some (Figure 5G). Colocalization of granules with F-actin was first measured as a percent of the granule area that contained an actin signal. There was 58±31% colocalization at the synapse, compared to 11±18% colocalization at a distance of 0.5–1 µm from the synapse, indicating that the granules were extensively contacting the actin network after they were delivered to the synapse. To further quantitatively analyze these interactions, we measured line profiles of intensity values for F-actin and perforin across the granule (Figure 5H). Granules were localized within minimally sized clearances, in slightly larger clearances, or directly atop filaments. In all cases, there was an association of the granule with actin as defined by granule and actin line profiles intersecting and/or overlapping. Thus granules use both minimally sufficiently sized clearances and only slightly larger clearances to gain access to the plasma membrane, and they do so in direct interaction with the actin network.

**Figure 6:**
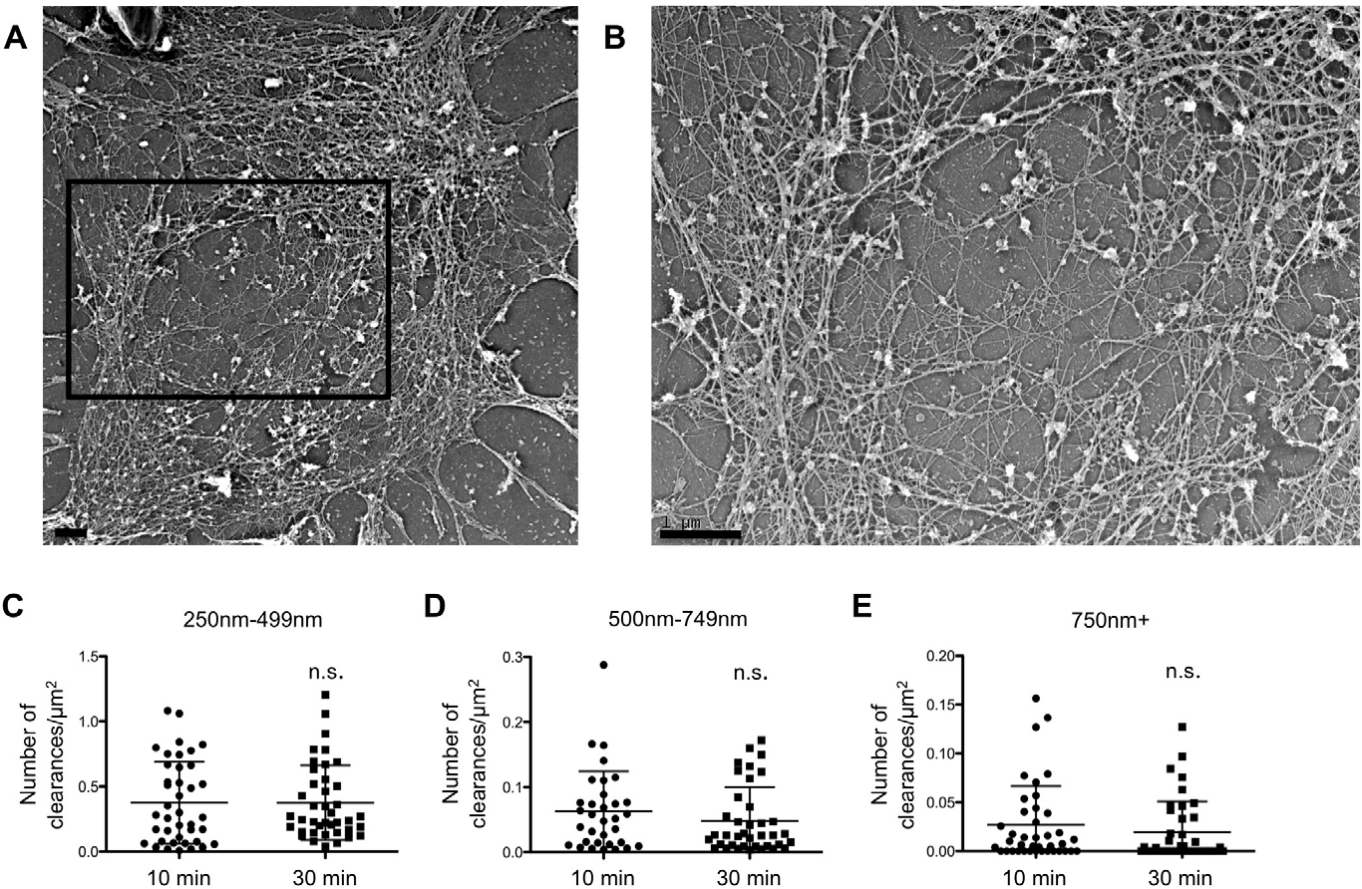
Platinum replica electron microscopy of the synapse further supports a network of actin filaments with lytic granule-sized clearances. NK-92 cells activated by immobilized antibodies to NKp30 and CD18 for 10 min or 30 min and then sheared apart by sonication were viewed by platinum replica electron microscopy. (A) Activated cortical synapse at 30 min of activation. (B) Enlargement of central boxed region in (A). Scale bars = 1 µm. (C–E) Measurements and comparison of the number of clearances of specified sizes: 250–499 nm (C), 500–749 nm (D), and greater than 750 nm (E).

To obtain unprecedented, nanometer resolution of actin filaments at the synapse, we used platinum replica electron microscopy. In order to expose the inner surface of cells, corresponding to the synapse, for metal coating, cells were “unroofed” by mechanical removal of the bulk of the cell body with the nucleus. Images of platinum replicas of the activated synapse confirmed our earlier light microscopy data that the F-actin network exists throughout the synapse and contains small granule-sized clearances (Figure 6A,B). Filaments within the network, however, were present in varying densities. Consistent with the requirement of WASp for synaptic maturation and previous studies defining the localization of the Arp2/3 complex to the synapse [9],[10], branched arrays of filaments were detected (Figure S11), although the branching frequency appeared lower, as compared to a typical branched network in lamellipodia, and many long filaments were also present.

In order to determine whether the abundance or distribution of clearances changes over time, the filamentous network at the synapse was evaluated after 10 and 30 min of activation. Quantitative assessment of the actin network in multiple cells defined a similar total contact area and total area occupied by filaments (filament density) between the two timepoints (Figure S12A,B). We also measured the individual groups of clearances in the actin network that were appropriately sized to allow lytic granule passage. We were able to detect clearances that would accommodate a granule at both timepoints (Figures 6C–E, S13). The number of clearances at each of the size thresholds was similar at the two activation timepoints tested. The distance of these clearances from the cell centroid, however, was different between the timepoints. After 30 min of activation the clearances were closer to the cell center than at 10 min (Figure S12C), suggesting that a fine-tuning of the actin network occurs and may be a necessary process in the maturation leading to degranulation. Thus ultra-resolution imaging of the synaptic actin network further defined the presence of minimally sufficiently sized clearances that would allow granule passage and demonstrated their changing position over time.

**Figure 7:**
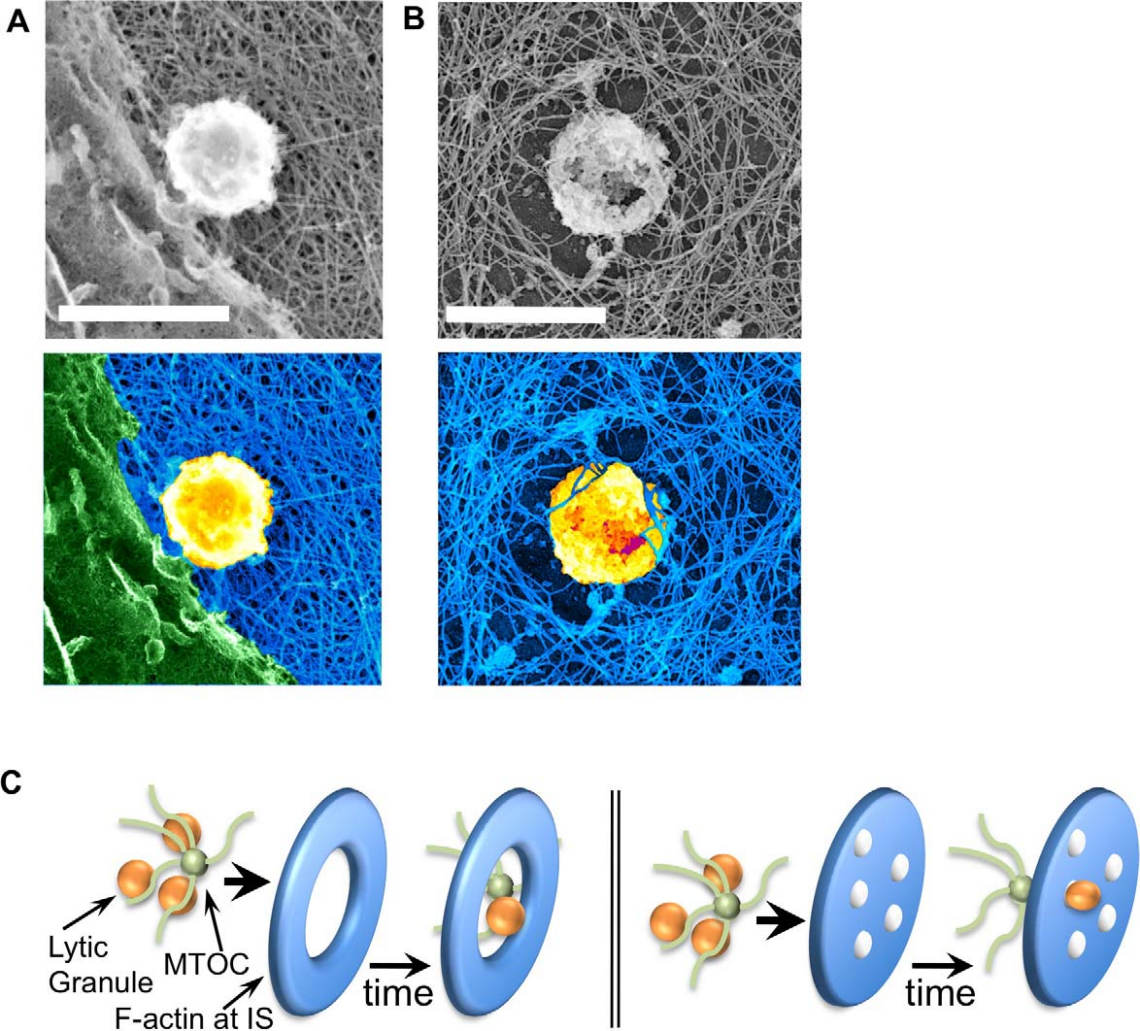
Approximation of lytic granule-sized organelles with and within cortical actin networks. (A,B) 20,000× images taken using platinum replica electron microscopy following activation of NK-92 cells by immobilized antibody to NKp30 and CD18 and then “unroofing” with nitrocellulose membrane (top) (scale bar = 1 µm). Pseudocolored images are shown (bottom) highlighting filaments (blue), granules (yellow), and plasma membrane (green). (C) Traditional (left) and proposed (right) model of granule delivery through the immunologic synapse.

Our TIRF data suggested, and our STED data demonstrated, that granules approximate the synapse in close association with the actin network. To confirm this observation on the nanometer level we used platinum replica electron microscopy to image “unroofed,” membrane-intact NK cells. Granule-sized organelles could be identified on the intracellular face of the actin network in approximation with filaments as well as intercalated within filament clearances (Figure 7A,B). These data are consistent with a model of granule approximation and degranulation whereby granules transit to the membrane through an interaction with the F-actin network and utilize multiple minimally sufficiently sized clearances instead of a single large opening (Figure 7C).

## Discussion

Actin accumulation defines an early stage in the maturation of the NK cell IS and is required for subsequent cytolytic function. Without proper reorganization of the actin cytoskeleton, lytic granules fail to polarize to the synapse and NK cells display inadequate cytotoxicity. Upon polarization, lytic granules require myosin IIA function to approximate the plasma membrane and have their contents directly secreted. The dependence on an actin motor protein for the secretory process suggests a requirement for the actin network itself. We have defined the distribution of actin at the IS using techniques that provide unprecedented sensitivity and resolution. We have done so both by live cells conjugated to target cells as well as a more flexible model system. We have also shown that lytic granules reach the plasma membrane and are secreted in areas of actin. Furthermore, our data suggest that secretion events likely occur in minimally yet sufficiently sized clearances in the actin network.

While our studies relied upon activating an NK cell line using immobilized antibody we were able to obtain physiologically relevant supporting data. Firstly, NK-92 cells activated in this manner released contents of lytic granules (Figure S1). Secondly, the distribution of actin at the IS in NK-92 cells was similar on immobilized antibody and on flat, living target cells even when using enhanced resolution (Figures 1B,C, S2A, 5A,C, 5G, 6A,B). Thirdly, to show direct ex vivo supporting evidence, the F-actin distribution at the IS of freshly isolated human NK cells was consistent with that seen in NK-92 cells (Figure 1). Thus the actin network we imaged using our model activation method was unlikely to be an artifact. A benefit of our model approach, however, was the ability to utilize platinum replica electron microscopy to image the synapse with nanometer resolution, and then correlate these findings with our fluorescence microscopy data.

The F-actin network in secretory cells was first believed to be a barrier to exocytosis [26]. This hypothesis was supported by data from numerous cell types including neuroendocrine cells (rev. in [27],[28]), neurons (rev. in [29]), platelets [30], and goblet cells [31] among others where loss of cortical F-actin correlated with an increase in secretion. In many of these cell types it has become increasingly appreciated that F-actin also serves as a required facilitator of secretion [27]–[29],[32]. In cells of the immune system, there is evidence for both models of secretion. Mast cells require some degree of F-actin present for agonist-induced secretion as robust depletion of F-actin with latrunculin interfered with normal release [33]. Conversely, F-actin is reported to be a barrier to secretion for neutrophil granule secretion [34].

Investigations with F-actin and granule secretion at the IS in cytotoxic lymphocytes have thus far been limited to T cells. In cytotoxic T lymphocytes, it has been suggested that actin is not a barrier to secretion [35],[36]. This includes the observation that actin is cleared from portions of the IS and granules are delivered to the synaptic membrane by the microtubule organizing center [35]. Because of 3-D confocal microscopy depicting F-actin rings at the NK cell IS [5],[11], the delivery of lytic granules is believed to occur in actin-devoid regions as in T cells. However, the reliance for granule secretion on myosin IIA in NK cells has called this model into question [12]. Specifically, that myosin IIA associates with lytic granules and its function is required for approximation to the synaptic membrane [13] strongly suggests that the mechanism for granule secretion is different in NK cells.

Our data support a role for F-actin as a facilitator of secretion rather than a barrier. Inhibiting actin polymerization with cytochalasin D or latrunculin A after activation resulted in diminished secretion of lytic granule contents rather than an increase. This suggested that actin was not a barrier to secretion, but that its dynamics were required. The latter hypothesis is supported by the results showing a similar inhibitory effect when cells were treated with jasplakinolide. Interestingly, the effect of the actin inhibitors was most pronounced at 10 min following activation and less so at 20 min following activation, and thus defined a critical window of actin reorganization that facilitates degranulation (Figure 4). One explanation for this is that reorganization is required for the bulk of granules to approach the membrane and dock, which is accomplished by 20 min. Regulation of actin reorganization as a requirement for secretion has been proposed in other cell types. In muscle cells the Arp2/3 complex and cofilin function in the required actin reorganization for GLUT4 vesicle exocytosis [37]. In chromaffin cells, Cdc42, neural-WASp, and the Arp2/3 complex are proposed to function together at the plasma membrane to facilitate secretion through generation of new filaments [38].

Our work defines an actin network that is more pervasive at the NK cell IS than previously thought. Although this could serve as a potential barrier, we have identified abundant granule-sized clearances that could function as sufficient access points to the plasma membrane. These could provide functionality by allowing granules to pass between filaments and to simultaneously interact with them, whereby myosin IIA could exert force in squeezing granules between filaments or in post-fusion expulsion of granule contents. This latter possibility has been suggested in chromaffin cells where myosin II function was required for appropriate release of catecholamines [39]. Another consideration is that granules may use clearances smaller than their equatorial area by squeezing through adjacent filaments. Although we cannot rule out this possibility, it is nevertheless a potential mechanism that is consistent with our hypothesis. Thus we propose that degranulation events at the NK cell IS represent a coupled interplay between actin filaments and clearances that presents additional opportunities for regulatory steps important to NK cell cytotoxicity.

## Materials and Methods

### Cell Lines and Ex Vivo NK Cells

NK-92 and GFP-actin expressing NK-92 cell lines were a kind gift from K. Campbell and were maintained in Myelocult (StemCell) media supplemented with 100 U/mL penicillin and streptomycin (Gibco) and 100 U/mL IL-2 (Hoffman-La Roche). mCherry-actin, Citrine-actin, and pHluorin-LAMP1 expressing cells were generated by retroviral transduction of NK-92 cells as described [40]. Briefly, 2–4 µg of plasmid DNA were transfected into the Phoenix packaging line using Fugene (Roche) lipofection reagent. Supernatant was harvested on day 2 post-transfection. NK-92 cells, Polybrene (Sigma), and supernatant were mixed and spun in a well of a 6 well plate at 1,000×g for 90 min at 32°C. Following overnight incubation at 32°C, cells were spun down and resuspended in supplemented Myeolocult. Cells were grown for 3 d prior to the introduction of puromycin (2 µg/mL) (InvivoGen) or hygromycin B (150 µg/mL) (Cellgro). mCherry-actin and Citrine-actin expressing cells were sorted for high expression by the University of Pennsylvania Cell Sorting Facility. Ex vivo NK cells were prepared from concentrated whole blood as described [41].

### Generation of Plasmids

The pHluorin-LAMP1 retroviral plasmid was generated by BioMeans, Inc. by inserting the sequence for pHluorin (a kind gift from G. Miesenböck) between the signal sequence and the transmembrane domain of IL-2Rα linked to the cytoplasmic tail of LAMP1 (a kind gift from M. Marks). A flexible GS linker was added between pHluorin and the transmembrane domain sequences. The entire construct was subsequently cloned into the MIGR1-puromycin vector.

The mCherry-actin retroviral plasmid was generated by PCR amplifying mCherry-actin from a pmCherry plasmid with 5′ BglII and 3′ EcoRI restriction site overhangs. The PCR product was digested and ligated into the pMSCV-Hygromycin plasmid (a kind gift from W. Pear), which had an EcoRI site in the Hygromycin resistance gene sequence eliminated by site-directed mutagenesis. The Citrine-actin retroviral plasmid was generated by amplifying the Citrine sequence from the pRSET-b Citrine plasmid (a kind gift from R. Tsien) with 5′ and 3′ BglII overhangs. The product was digested and ligated into a similarly digested mCherry-actin retroviral plasmid, effectively removing the mCherry sequence and inserting the Citrine sequence. Proper orientation of insert was verified by DNA sequencing by the Children's Hospital of Philadelphia Research Institute sequencing core facility.

### Flow Cytometry

Flow cytometry was performed to verify pHluorin-LAMP1 expression. Cells were untreated or treated with phorbol myristate acetate (PMA, 100 ng/mL, Sigma) and Ionomycin (1 µg/mL, Sigma) for 30 min or Concanamycin A (CMA, 100 nM, Sigma) for 90 min and samples were run on a BD FACSCalibur.

### Total Internal Reflection Fluorescence Microscopy

Cells were washed and resuspended in supplemented Myelocult prior to use. For imaging of lytic granules, cells were incubated with 100 nM LysoTracker Red DND-99 (Molecular Probes) for 30 min at 37°C, washed once, and resuspended in supplemented Myelocult. ΔT dishes (Bioptechs) were coated with 5 µg/mL anti-NKp30 (Beckman-Coulter) and 5 µg/mL anti-CD18 (Clone IB4) for 1 h at 37°C, washed with PBS, and prewarmed prior to imaging with 1 mL dye free R10 (dye free RPMI 1640 (Gibco), 10% fetal bovine serum (Atlanta Biologicals), 10 mM HEPES (Gibco), 100 U/mL penicillin and streptomycin, 100 µM MEM nonessential amino acids (Gibco), 1 mM sodium pyruvate (CellGro), and 2 mM L-glutamine (Gibco). 4×10^5^ cells were added to the dishes, which were maintained at 37°C with a heated stage and lid (Bioptechs).

For live cell imaging of actin dynamics following inhibitor treatment, cells were activated as above for 10 min before addition of media containing DMSO or jasplakinolide (1 µM, Calbiochem). Following 5 min of incubation, media containing DMSO or latrunculin A (10 µM, Sigma) was added to the dish. After 5 min of further incubation, cells were imaged.

For fixed cell experiments, 1×10^5^ cells were adhered to No. 1 glass coverslips coated with antibody as described above. Samples were fixed and stained with Alexa Fluor 488 phalloidin or 568 phalloidin (Molecular Probes) as described [41]. For experiments with inhibitor treatments, cells were activated on coverslips for 10 min or 20 min, treated with inhibitors for 5 min, and then fixed and stained. Cytochalasin D (Sigma) was used at 10 µM.

Samples were imaged through a 1.49 NA, oil immersion, 60×, APO N TIRFm objective or a 1.45 NA, oil immersion, 100×, PlanApo TIRFm objective (Olympus) when noted. 488 nm (Spectra-Physics) and 561 nm (Cobalt) diode lasers were launched through a two-line combiner of an LMM5 (Spectral Applied Research) into a rear mounted TIRF illuminator (Olympus) on an Olympus IX-81. Lasers were aligned for total internal reflection prior to each experiment. Images were captured using Volocity (PerkinElmer) to control a C9100 EM-CCD camera (Hamamatsu).

### Confocal Microscopy

Mel1190 cells were plated into ΔT dishes 1 d prior to use. Cells were stained with CellMask Deep Red (Invitrogen) according to manufacturer's instructions just prior to imaging. GFP-actin expressing NK-92 cells were added to the dishes, which were maintained at 37°C, and imaged for up to 1 h. Cells were imaged through a 63×1.4 NA Plan-APOCHROMAT objective (Zeiss) on a Zeiss Observer.Z1 using a C10600 ORCA-R2 camera (Hamamatsu). The microscope was equipped with a CSU10 spinning disk system (Yokogawa). 491 nm (Cobalt) and 655 nm (CrystaLaser) diode lasers were launched through an LMM5 (Spectral Applied Research).

NK-92 cells were activated on antibody coated glass coverslips as described above for 30 min and then fixed and stained with rabbit anti-human Pericentrin (Abcam), Alexa Fluor 488-phalloidin, and anti-Perforin-Alexa Fluor 647 (Biolegend). The secondary antibody to anti-Pericentrin was a goat anti-mouse Pacific Blue (Molecular Probes). Cells were imaged in three dimensions on a spinning disk confocal Olympus DSU IX-81 microscope.

### Stimulated-Emission Depletion (STED) Microscopy

Mel1190 cells were grown in a monolayer overnight on No. 1.5 glass coverslips. Citrine-actin expressing NK-92 cells (10^6^) were resuspended in media and incubated on Mel1190 targets for 30′ at 37°C. Cells were fixed with 2% paraformaldehyde and mounted with ProLong antifade reagent (Invitrogen). Cells were imaged at the plane of the interface between NK and target cells. Separately, for imaging of granules, Citrine-actin expressing NK-92 cells were immobilized to bound antibody as described above, then fixed, permeabilized, and stained with anti-perforin Alexa Fluor 488 (Biolegend).

For visualization of actin and perforin in NK-92 cells, cells were immobilized to bound antibody as described above, then fixed, permeabilized, stained with Alexa Fluor 488 phalloidin and anti-perforin Alexa Fluor 647, and imaged at the plane of the glass or 0.5–1 µm above it.

All samples were mounted with Prolong anti-fade reagent (Invitrogen). Cells were imaged through a 100×1.4 NA HCX APO objective on a Leica TCS STED CW system controlled by Leica AS AF software. Alexa Fluor 488 and Citrine were excited using a 488 nm Argon laser and STED depletion was achieved using a 592 nm continuous wave fiber laser. Alexa Flour 647 was excited using a HeNe 633 laser and imaged using the laser scanning confocal modality of the system. Fluorescence was detected with HyD detectors (Leica).

### Platinum Replica Electron Microscopy

Cells were washed, resuspended in supplemented Myelocult, and allowed to adhere to 0.15 glass coverslips coated with antibody as described above. For imaging of the actin network alone, samples were prepared following a modified protocol described in [42]. After a period of incubation at 37°C, samples were washed once in PBS, dipped into a 1∶3 dilution of PEM buffer (0.1 M PIPES (Sigma), pH 6.9, 1 mM EGTA (Sigma), 1 mM MgCl_2_ (Sigma)) in dH_2_0 for 10 s, sonicated in PEM for 1–2 s at a 45° angle to the probe, and incubated with 1% Triton X-100 (Sigma) in PEM for 1–2 min before fixation in 2% glutaraldehyde (Fluka) in PEM. For imaging of granules, cells were “unroofed” by applying a nitrocellulose membrane that had been wet in PEM to the coverslip for 45–60 s, removing it, and then fixing the sample in 2% glutaraldehyde in PEM. All samples were processed for EM as described [43]. Platinum replicas were imaged on a JEM 1011 transmission electron microscopy (JEOL USA) at 100 kV. Images were captured using an ORIUS 835.10W CCD camera (Gatan) and are presented in inverted contrast.

### Degranulation Assay

Immulon 4HBX 96 well flat bottom plates (Thermo) were coated with murine IgG (BD), anti-human NKp30, anti-human CD18, or both anti-NKp30 and anti-CD18 at 5 µg/mL in PBS overnight at 4°C. Plates were washed twice with PBS and blocked for 1 h at room temperature with R10 media. 1×10^5^ cells were added to wells and plates were spun at 1,000 rpm for 2 min before incubation at 37°C. For the timecourse degranulation assay, supernatants from spontaneous and activated wells were harvested at indicated times. For inhibitor treatments, media containing inhibitor or vehicle (DMSO) was added at indicated times. Thapsigargin (Calbiochem) was used at 1 µM. Supernatants were harvested after 60 min. For total release, cells were lysed in 0.5% Nonidet P40 (Accurate Chemical and Scientific).

Supernatants were assayed by mixing 20 µL supernatant with 200 µL of a solution containing PBS, 9.8 mM HEPES (Gibco), 196 µM Z-L-Lys-SBzl hydrochloride (BLT, Sigma), and 218 µM 5,5′-dithiobis(2-nitrobenzoic acid) (DTNB, Sigma). Samples were incubated for 30 min at 37°C and absorbance was measured immediately at 405 nm. Percent total release was measured by subtracting the spontaneous release value from activated release values (A–S) and the total release value (T–S), and then dividing (A–S) by (T–S).

### Image Analysis

Images and timelapse series were analyzed using either Volocity or the FIJI package of ImageJ (http://pacific.mpi-cbg.de). Using Volocity, fluorescently tagged actin footprints were identified using a classifier that identifies objects above a selected standard deviation above the mean intensity (usually 0–1). LysoTracker and pHluorin positive events were similarly identified (4–10 standard deviations above mean intensity), with the additional exclusion of events smaller than 0.05 µm^2^. For actin fluorescence ratio measurements, the MFI at the location of granule approximation or degranulation was divided by the MFI of the actin footprint. Maximum and minimum potential actin intensities were also measured by dividing the maximum and minimum pixel intensities of the actin footprint by the MFI of the footprint. For the “adjacent points” measurement, the region of interest (ROI) that was identified for the degranulation (pHluorin) signal was moved to four neighboring locations. The actin intensities were measured, averaged, and then divided by the MFI of the actin footprint to generate a single value.

Distance of granules from the MTOC was determined as described [17] by inputting the coordinates of the MTOC centroid and a granule centroid into the Pythagorean equation (a^2^+b^2^ = c^2^: (X_centroid_−X_clearance_)^2^+(Y_centroid_−Y_clearance_)^2^ = c^2^, where c is the distance from the MTOC).

FIJI was used to generate radial intensity profiles using the radial profile plugin (http://rsbweb.nih.gov/ij/plugins/radial-profile.html). For a profile of the entire cell footprint an ROI circle was drawn around the cell and data from running the plugin were exported to Excel (Microsoft). For profiles of degranulation events images were first split into red and green images and then a circle with a radius of 8 pixels (1.08 µm) was drawn around each event. Data were generated for 6 radii from the center of each event. To determine the radial intensity change, an outer radial intensity value was subtracted from an inner radial intensity value. Thus a negative value indicates that the outer circle has higher mean intensity than the inner circle.

To measure changes in the actin network over time, sequential images were imported into FIJI, a line was drawn across the center of the cell, and line intensity profile data were generated for each time point at the same location within the cell. Data were exported to Excel and standard deviations calculated. Surface plots were generated using the surface plot function in FIJI.

Images of granules taken using STED microscopy were analyzed in Volocity and diameters measured by drawing lines across the center of the granules. STED microscopy images of the actin network were imported into FIJI and processed before analysis. Background was subtracted using the Rolling Ball Subtraction algorithm with the radius set to 150 pixels and then pixel intensities were squared twice. An ROI was drawn around the interior of the cell and clearances were identified using the default autothreshold with “dark background” unchecked. Clearance areas were sorted, grouped, and counted in Excel based on size. Dividing the number of clearances per cell by the area measured normalized the values. Area cutoffs were implemented by using granule diameter as a reference. The smallest two sizes calculated assume that granules are uniformly spherical and require a clearance that has an area that would accommodate the equatorial area of the granule. The larger size categorizes all clearances that are larger than most granules.

Colocalization analysis was performed in Volocity using the “Find objects” tool to identify granules and a fixed intensity threshold, which was adjusted to generate interfilament or intercellular black space on a per field basis, to identify the actin network. The colocalized area of granule and actin network staining was divided by the area of the granule to yield a value denoting the percent of the granule area colocalized with actin. Five granules from 10 cells over 2 experiments were measured (*N* = 50 granules). Line profiles of granules and the actin network were performed in FIJI after channels were separated. All STED images were measured in their raw form, but shown after processing in Leica AS AF with the median noise reduction feature.

Images from platinum replica electron microscopy were inverted and linearly contrast enhanced using Photoshop (Adobe) and imported into FIJI for processing and analysis. Background was subtracted from each image using the Rolling Ball Subtraction algorithm with the radius set to 25 pixels. Pixel intensities were subsequently squared. Cells were identified using the default autothreshold and cell contact area and cell centroid were measured with “include holes” checked. ROIs were drawn around the interior of the cell to more accurately identify filaments and to avoid debris. Filaments were identified using the default automatic threshold with “dark background” checked. Clearances in the filamentous network were identified as mentioned above. Distance from the cell centroid was determined by inputting the coordinates of the cell centroid and a clearance centroid into the Pythagorean equation as described above for granule to MTOC distance.

All data were plotted using Prism (Graphpad).

### Statistical Analyses

Statistical significance was determined using Prism to perform one- or two-sample, unpaired or paired, two-tailed Student's *t* tests. Unless otherwise indicated all tests were two-sample, two-tailed, and unpaired. Where noted, n.s. denotes differences that have *p* values >0.05 and therefore considered not significant.

## Supporting Information

Figure S1 Timecourse of degranulation of activated NK-92 cells. NK-92 cells were activated by immobilized antibody to NKp30 and CD18 and incubated at 37°C. (A) Supernatants were harvested at indicated times and assayed for Granzyme A activity using the BLT esterase assay and results are shown as a percent of total potential release. Single antibody control supernatants were harvested following 60 min of activation. Values shown represent the mean + SD of three independent experiments.
(TIF)

Figure S2 Kinetics and sustenance of actin accumulation at the activated IS. (A) GFP-actin (green) expressing NK-92 cells were activated on immobilized antibody to NKp30 and CD18 and imaged by TIRF microscopy. Images were acquired over 50 min at a rate of 1 frame per minute. Images of a representative cell are shown at 5-min intervals beginning following 2.5 min of contact. Scale bar = 5 µm. (B) Area and mean fluorescence intensity (MFI) for 6 cells plotted over time (error bars, ± SD). Data are representative of three independent experiments.
(TIF)

Figure S3 Actin hypodensities present within the NK cell synaptic cortex. NK-92 cells expressing GFP-actin were activated and imaged by TIRFm. (A) Image of GFP-actin at 30 min post-activation. Scale bar = 5 µm. (B) Magnification of boxed region from (A). (C) Line profile of intensity from dotted line in (B).
(TIF)

Figure S4 Quantitative analysis of granule approximation to the actin network. (A) The intensity of GFP-actin fluorescence at the point of granule approximation was determined by dividing the MFI of GFP-actin in the granule region by the MFI of the GFP-actin of the whole cell in the TIRF field. This yielded a ratio of MFI signals. Each point represents one granule. (B) Minimum (min) and maximum (max) possible values are plotted along with the mean. Min and max values were determined by using the minimum and maximum pixel values of the GFP-actin signal in the TIRF field and the equation described in (A). (C) GFP ratio values for 14 cells plotted as in (A).
(TIF)

Figure S5 Model and implementation of pHluorin-LAMP1 construct. (A) Model of the construct depicting relative locations of sequences: endoplasmic reticulum targeting signal sequence (SS), flexible glycine-serine linker (GS), transmembrane domain (TM). (B) Diagram depicting fluorescent state of pHluorin depending on intralumenal versus surface location.
(TIF)

Figure S6 Degranulation events are less abundant than granule approximations. (A) pHluorin-LAMP1 expressing cells were loaded with LysoTracker Red and imaged for approximately 60 min at a rate of 1 frame per minute. (B) To count events, all frames from the acquisition were merged into a single image. (C) Number of Lysotracker positive and pHluorin positive events for each cell are plotted (*n* = 27; *** *p*<0.0001, paired *t* test).
(TIF)

Figure S7 Degranulation MFI actin ratios plotted relative to minimum and maximum potential ratios. MFI ratio of actin intensities at the point of degranulation to that of the respective footprints (black) is plotted relative to minimum (blue) and maximum (red) potential values for 52 events.
(TIF)

Figure S8 Radial intensity profile plots for synaptic actin following treatment with actin inhibitors. NK-92 cells were activated for 10 (A) or 20 min (B) before addition of DMSO or inhibitor. Following 5 min of incubation, cells were fixed, stained for actin with phalloidin, and imaged by TIRFm using a 100× objective. Radial intensity profiles were generated and averaged for 30 cells/condition over 3 experiments. For latrunculin A treated cells, DIC images were used for spatial reference since actin fluorescent signal was undetectable.
(TIF)

Figure S9 The actin network is dynamic at early and late timepoints of activation. GFP-actin expressing NK-92 cells were activated and imaged at a rate of 2 frames per minute after 10 min and 30 min of activation for 5 min. Scale bar = 5 µm. (A) Images from the first 2.5 min of the 10 to 15 min timeframe are shown. (B) Corresponding intensity surface plots from the timepoints shown in (A). Overlay of line profiles through the centroid of the cell contact from images taken between 10 and 15 min of activation (C), or 30–35 min (D) of activation. (E) To compare variation in multiple cells (*n* = 10) between the two timeframes, the standard deviation of mean intensity over 5 min for each pixel along the measured line was calculated for each cell. The mean standard deviation for each cell was calculated and plotted for the 10–15 min and 30–35 min timeframes. (F) The standard deviation of pixel intensity change over a stationary line was calculated as in (E) for at least 10 cells from 2 experiments following DMSO or sequential jasplakinolide and latrunculin A treatment after 10 min of activation (*** *p*<0.0001).
(TIF)

Figure S10 Diameters of granules imaged by STED microscopy and their relation to clearance area. (A) NK-92 cells were activated on glass, fixed, and stained for perforin. 104 granules were measured. (B) The mean clearance area for each cell (defined as any area large enough to accommodate a 250 nm in diameter granule) was divided by the mean granule equatorial area derived from (A) and plotted according to interval. The mean granule diameter of 333 nm corresponds to a mean equatorial area of 0.0871 µm^2^.
(TIF)

Figure S11 Branched networks at the activated IS. (A) High magnification image of filaments at the activated synapse using platinum replica electron microscopy. (B) Image from (A) with pseudocolored region indicating examples of branching filaments. Scale bar = 100 nm.
(TIF)

Figure S12 Additional analyses of cells imaged by platinum replica electron microscopy. (A–C) Comparative measurements of the synapse include: contact area (A); filament density (B); and distance from the cell centroid of individual clearances that would be greater than or equal in size to the equatorial area of a 250 nm granule (C) (* *p*<0.05, unpaired *t* test).
(TIF)

Figure S13 Algorithm-based identification of clearances in the F-actin network. Colored regions indicate appropriately sized clearances that were identified.
(TIF)

Video S1 Live cell imaging of activated GFP-actin expressing NK-92 cells. Cells were imaged at 2 frames per minute and are shown at 6 frames per second starting at time zero relative to activation.
(MOV)

Video S2 Live cell imaging of activated GFP-actin expressing, LysoTracker Red loaded NK-92 cells. Cells were imaged at 1 frame per minute and are shown at 4 frames per second starting 2 min after activation.
(MOV)

Video S3 Live imaging of a Lysotracker Red loaded granule in a pHluorin-LAMP1 expressing cell undergoing degranulation and attaining pHluorin fluorescence. Images were acquired at 2 frames per minute and are shown at 6 frames per second.
(MOV)

Video S4 Live cell imaging of activated pHluorin-LAMP1 expressing, LysoTracker Red loaded NK-92 cells. Cells were imaged at 1 frame per minute and are shown at 4 frames per second starting at time zero relative to activation. LysoTracker Red events outnumber pHluorin events.
(MOV)

Video S5 Live cell imaging of activated pHluorin-LAMP1 and mCherry-actin expressing NK-92 cells. Cells were imaged at 6 frames per minute and are shown at 6 frames per second. Video begins 25 min after activation.
(MOV)

Video S6 Live cell imaging and intensity surface plots of activated GFP-actin expressing NK-92 cells. Cells were imaged at 2 frames per minute and are shown at 2 frames per second starting at 10 min after activation.
(AVI)

## Abbreviations

CMA: concanamycin A
CW: continuous wave
F-actin: filamentous-actin
IS: immunological synapse
LAMP1: lysosomal-associated membrane protein 1
MFI: mean fluorescence intensity
MTOC: microtubule organizing center
NK: natural killer
PMA: phorbol myristate acetate
STED: stimulated emission depletion
TIRFm: total internal reflection fluorescence microscopy
WAS: Wiskott-Aldrich Syndrome
WASp: Wiskott-Aldrich Syndrome protein
