# Supplementary figures and images for: Natural Killer Cell Lytic Granule Secretion Occurs through a Pervasive Actin Network at the Immune Synapse
Source: PLoS Biol. 2011 Sep 13;9(9):e1001151. doi: 10.1371/journal.pbio.1001151 (PMC3172191; doi:10.1371/journal.pbio.1001151)

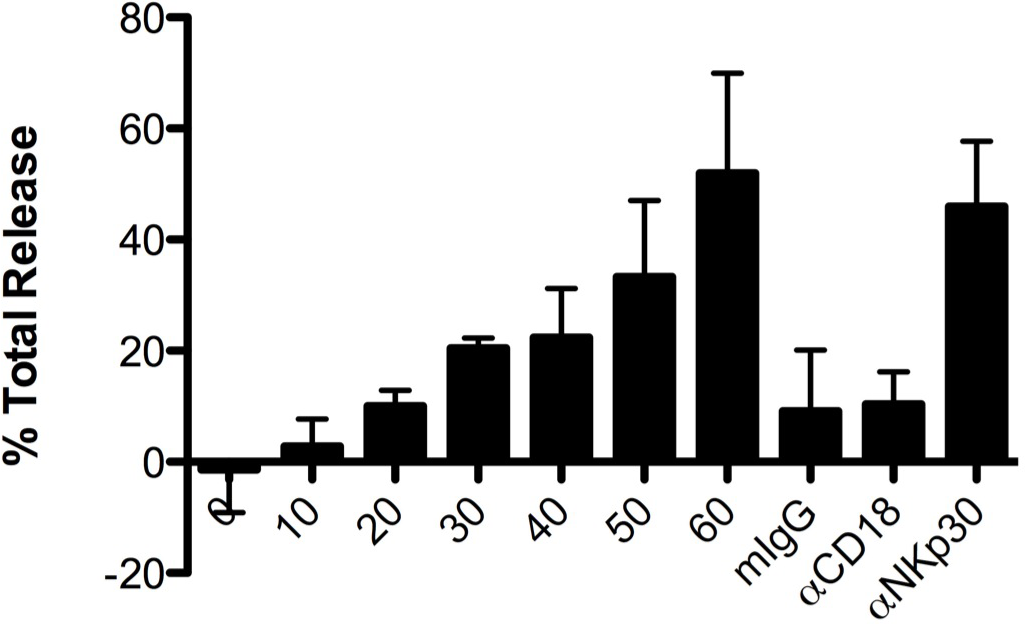

Supplement: Figure S1 — Timecourse of degranulation of activated NK-92 cells. NK-92 cells were activated by immobilized antibody to NKp30 and CD18 and incubated at 37°C. (A) Supernatants were harvested at indicated times and assayed for Granzyme A activity using the BLT esterase assay and results are shown as a percent of total potential release. Single antibody control supernatants were harvested following 60 min of activation. Values shown represent the mean + SD of three independent experiments. (TIF) [file pbio.1001151.s001.tif]

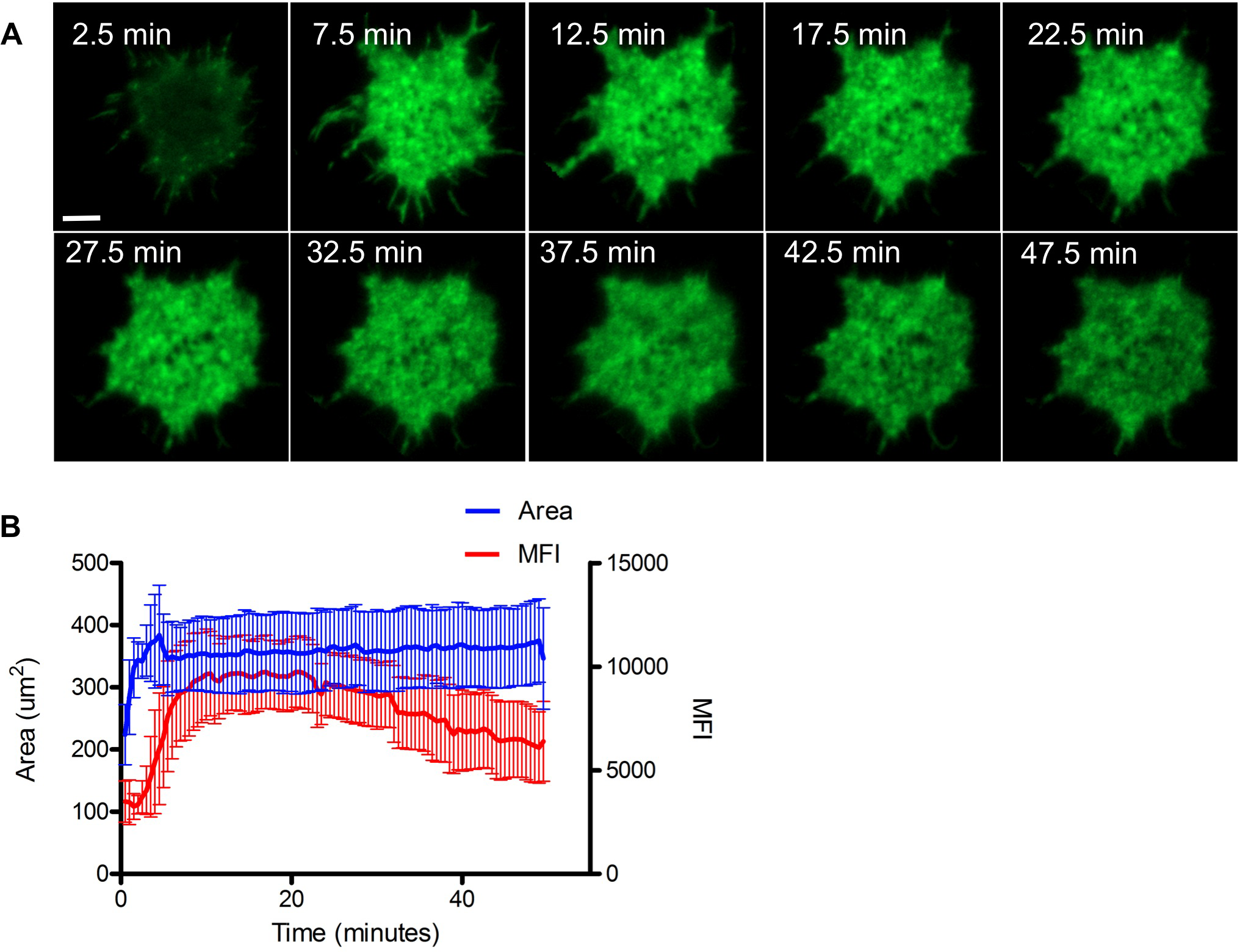

Supplement: Figure S2 — Kinetics and sustenance of actin accumulation at the activated IS. (A) GFP-actin (green) expressing NK-92 cells were activated on immobilized antibody to NKp30 and CD18 and imaged by TIRF microscopy. Images were acquired over 50 min at a rate of 1 frame per minute. Images of a representative cell are shown at 5-min intervals beginning following 2.5 min of contact. Scale bar = 5 µm. (B) Area and mean fluorescence intensity (MFI) for 6 cells plotted over time (error bars, ± SD). Data are representative of three independent experiments. (TIF) [file pbio.1001151.s002.tif]

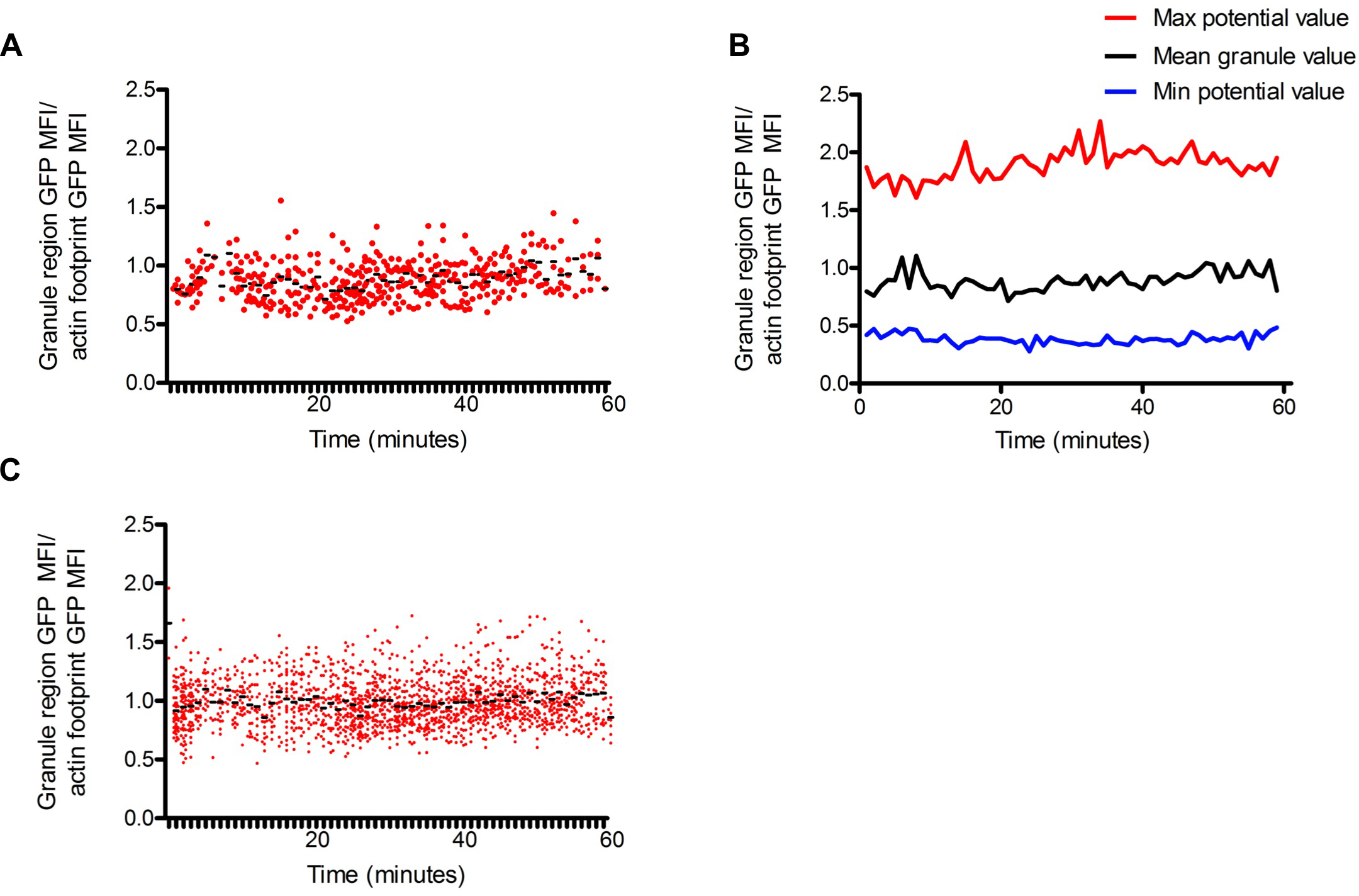

Supplement: Figure S3 — Actin hypodensities present within the NK cell synaptic cortex. NK-92 cells expressing GFP-actin were activated and imaged by TIRFm. (A) Image of GFP-actin at 30 min post-activation. Scale bar = 5 µm. (B) Magnification of boxed region from (A). (C) Line profile of intensity from dotted line in (B). (TIF) [file pbio.1001151.s003.tif]

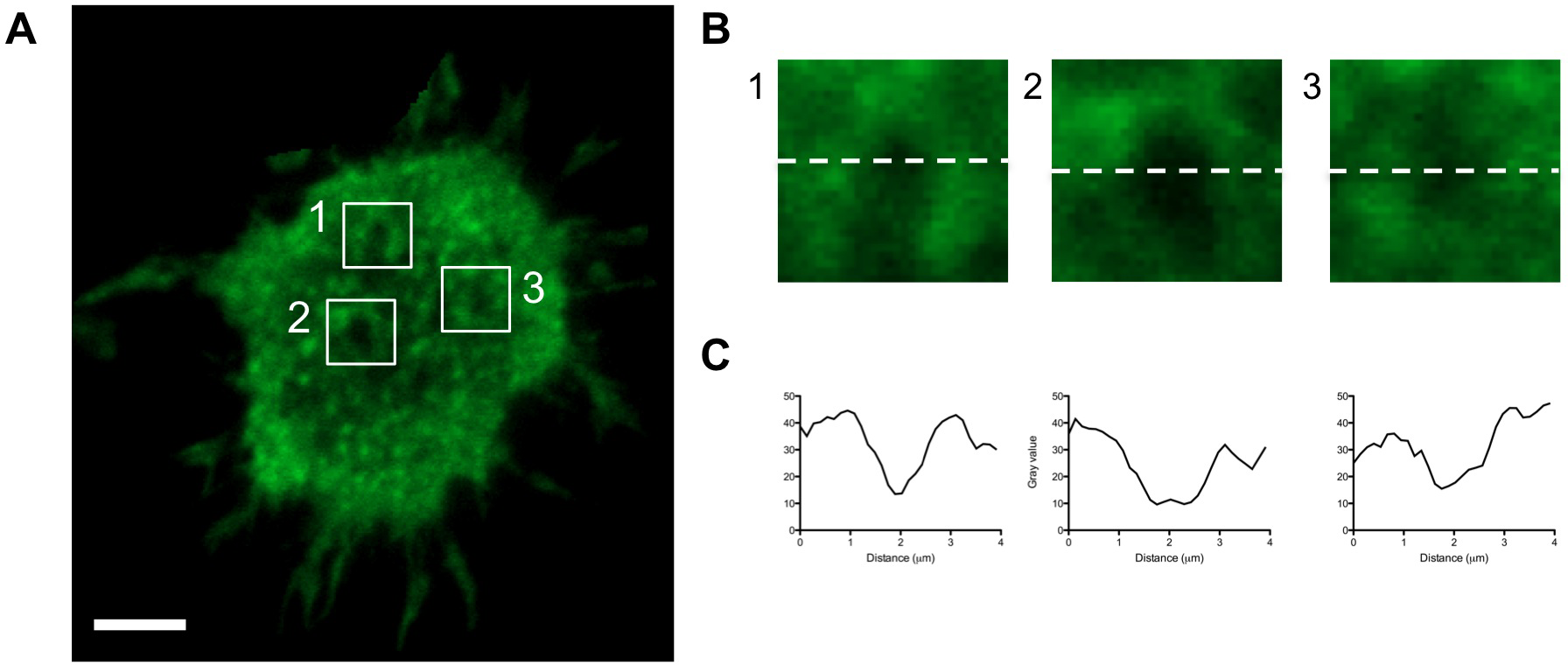

Supplement: Figure S4 — Quantitative analysis of granule approximation to the actin network. (A) The intensity of GFP-actin fluorescence at the point of granule approximation was determined by dividing the MFI of GFP-actin in the granule region by the MFI of the GFP-actin of the whole cell in the TIRF field. This yielded a ratio of MFI signals. Each point represents one granule. (B) Minimum (min) and maximum (max) possible values are plotted along with the mean. Min and max values were determined by using the minimum and maximum pixel values of the GFP-actin signal in the TIRF field and the equation described in (A). (C) GFP ratio values for 14 cells plotted as in (A). (TIF) [file pbio.1001151.s004.tif]

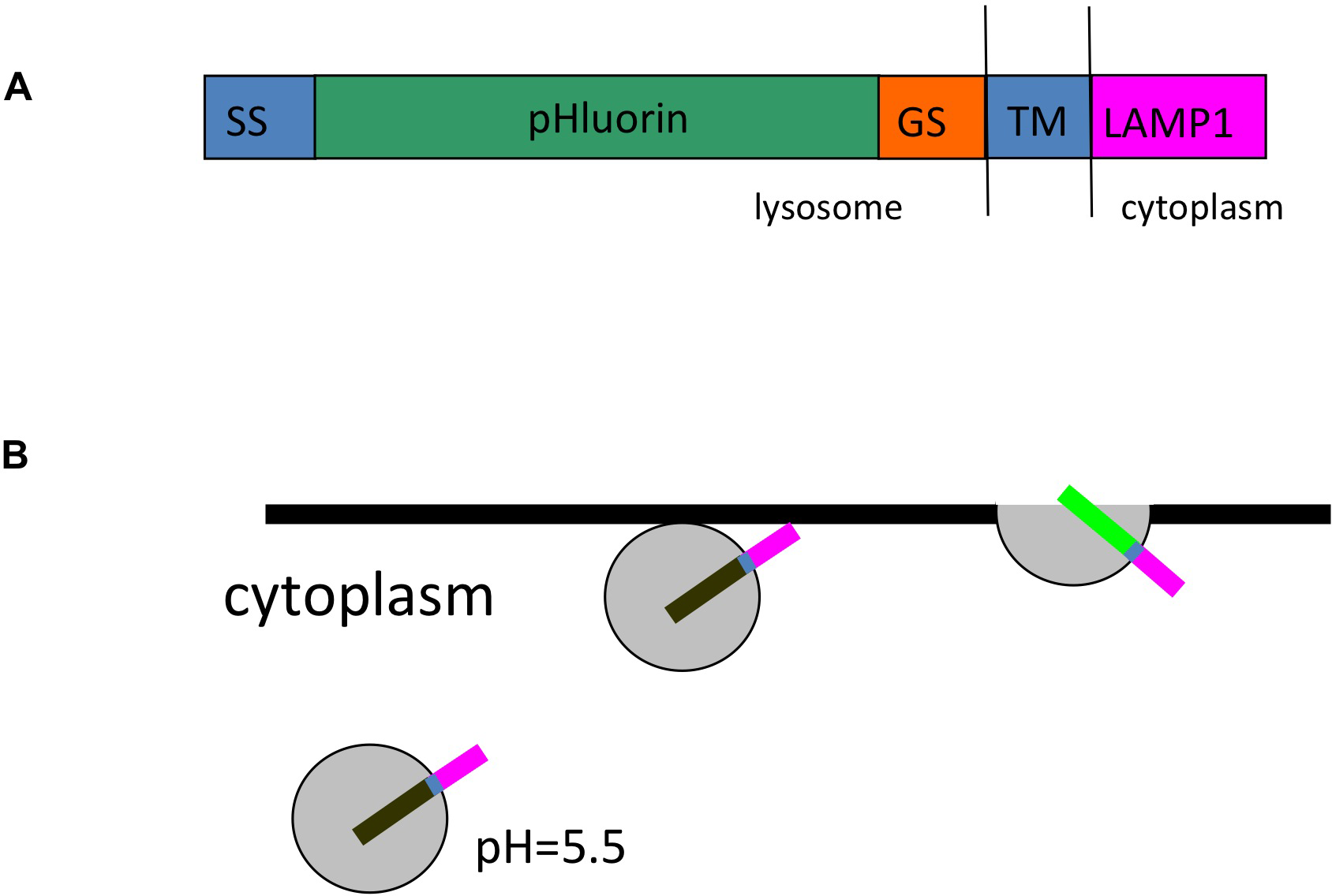

Supplement: Figure S5 — Model and implementation of pHluorin-LAMP1 construct. (A) Model of the construct depicting relative locations of sequences: endoplasmic reticulum targeting signal sequence (SS), flexible glycine-serine linker (GS), transmembrane domain (TM). (B) Diagram depicting fluorescent state of pHluorin depending on intralumenal versus surface location. (TIF) [file pbio.1001151.s005.tif]

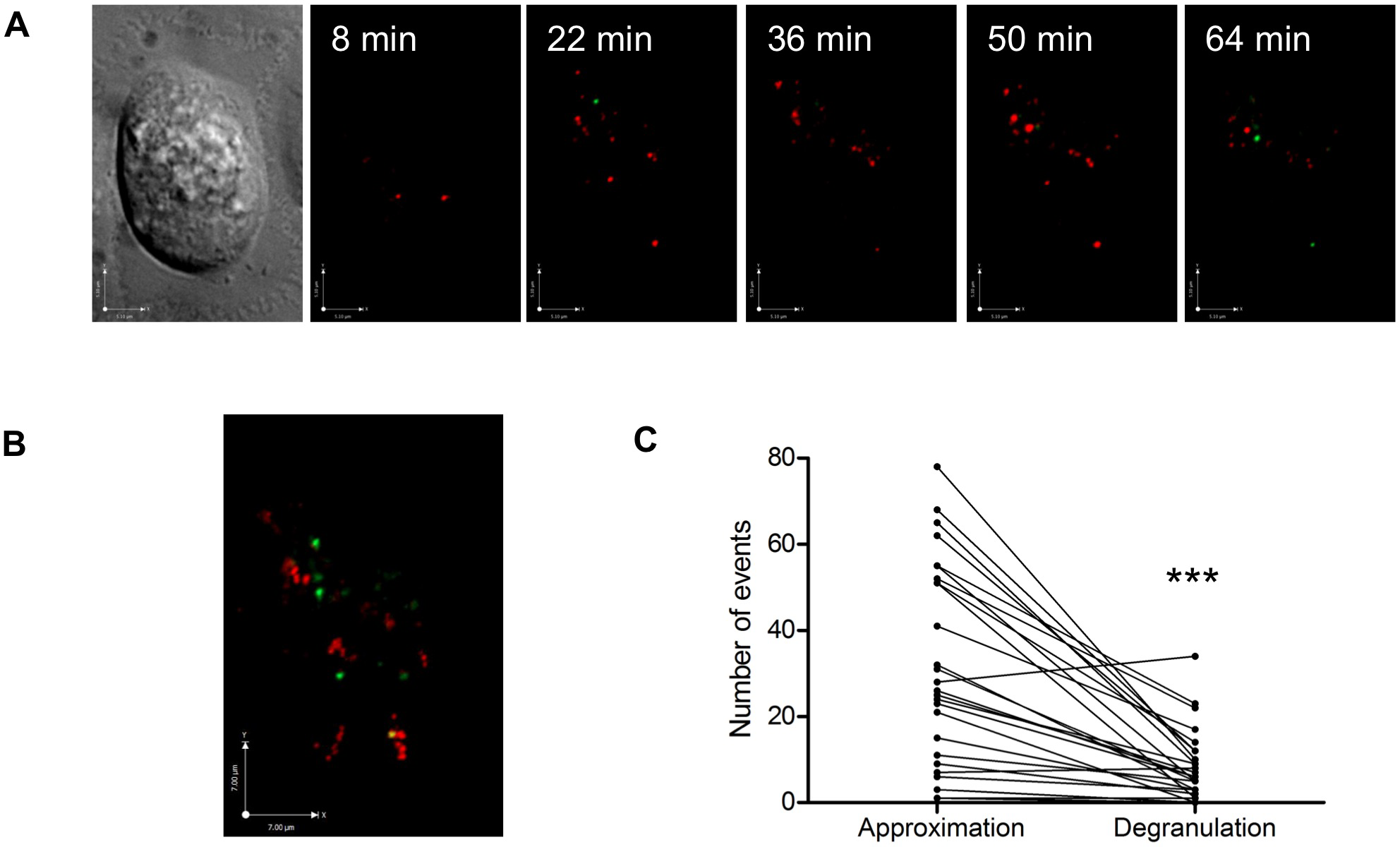

Supplement: Figure S6 — Degranulation events are less abundant than granule approximations. (A) pHluorin-LAMP1 expressing cells were loaded with LysoTracker Red and imaged for approximately 60 min at a rate of 1 frame per minute. (B) To count events, all frames from the acquisition were merged into a single image. (C) Number of Lysotracker positive and pHluorin positive events for each cell are plotted (n = 27; *** p<0.0001, paired t test). (TIF) [file pbio.1001151.s006.tif]

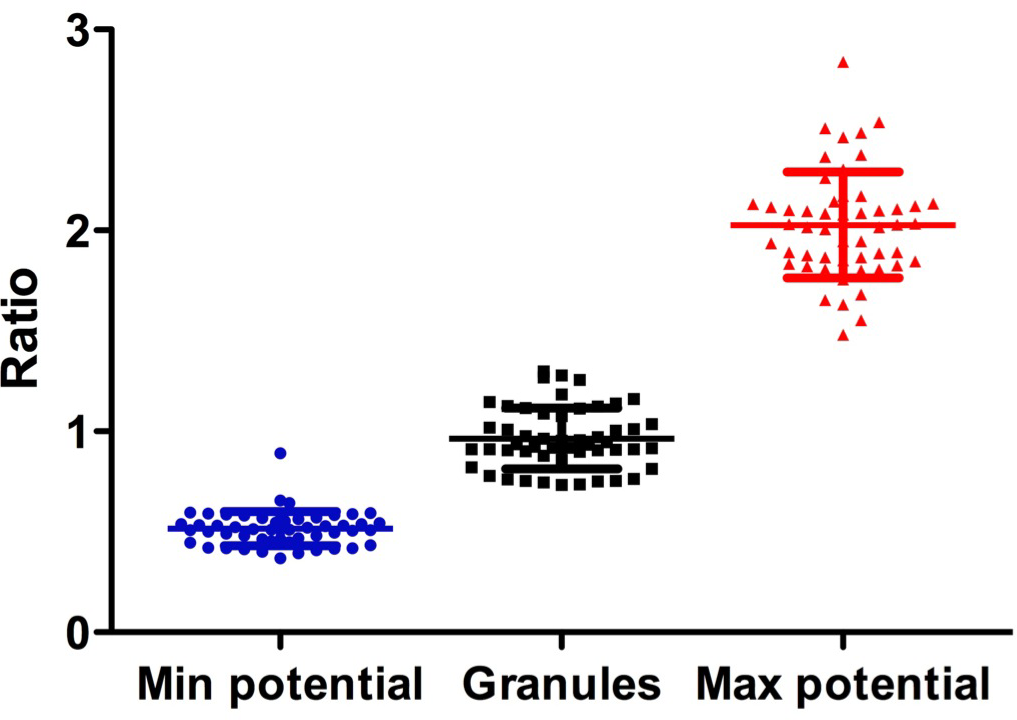

Supplement: Figure S7 — Degranulation MFI actin ratios plotted relative to minimum and maximum potential ratios. MFI ratio of actin intensities at the point of degranulation to that of the respective footprints (black) is plotted relative to minimum (blue) and maximum (red) potential values for 52 events. (TIF) [file pbio.1001151.s007.tif]

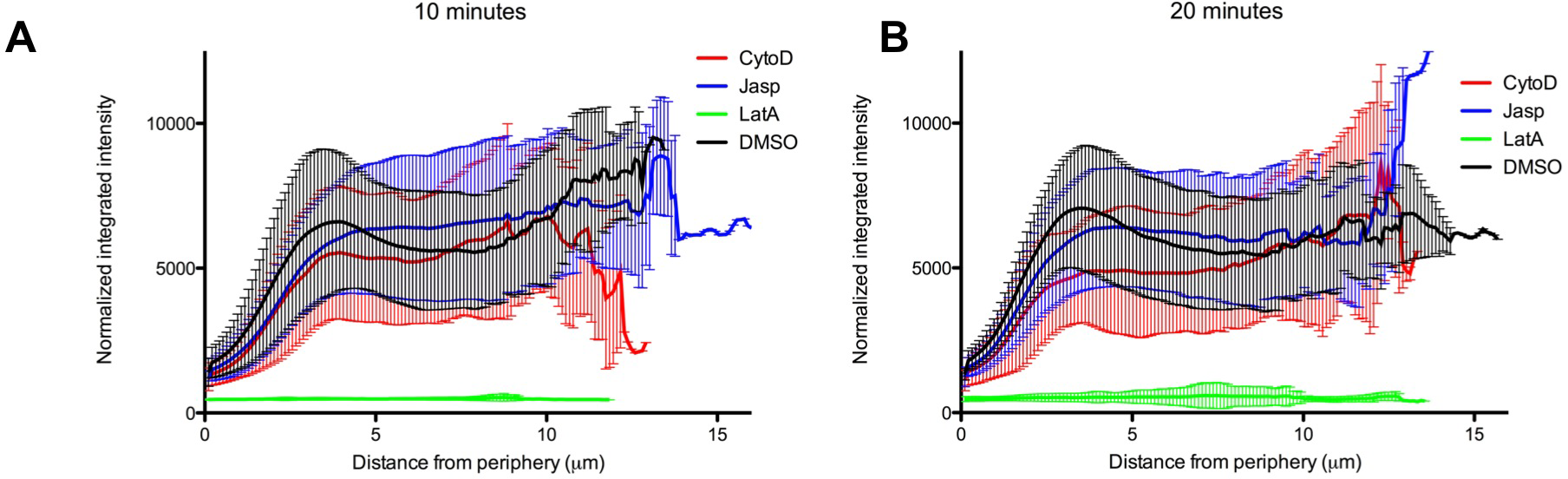

Supplement: Figure S8 — Radial intensity profile plots for synaptic actin following treatment with actin inhibitors. NK-92 cells were activated for 10 (A) or 20 min (B) before addition of DMSO or inhibitor. Following 5 min of incubation, cells were fixed, stained for actin with phalloidin, and imaged by TIRFm using a 100× objective. Radial intensity profiles were generated and averaged for 30 cells/condition over 3 experiments. For latrunculin A treated cells, DIC images were used for spatial reference since actin fluorescent signal was undetectable. (TIF) [file pbio.1001151.s008.tif]

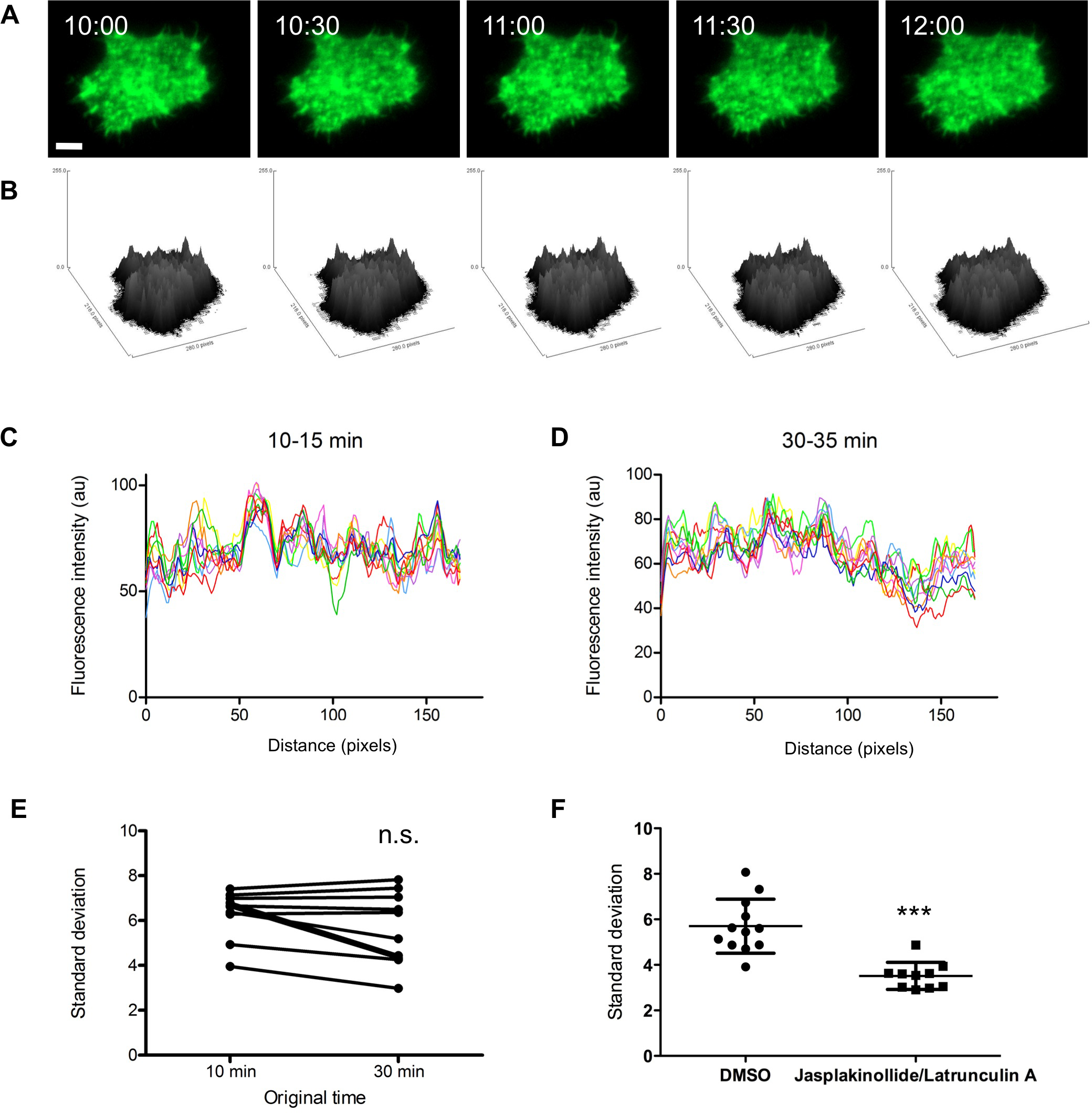

Supplement: Figure S9 — The actin network is dynamic at early and late timepoints of activation. GFP-actin expressing NK-92 cells were activated and imaged at a rate of 2 frames per minute after 10 min and 30 min of activation for 5 min. Scale bar = 5 µm. (A) Images from the first 2.5 min of the 10 to 15 min timeframe are shown. (B) Corresponding intensity surface plots from the timepoints shown in (A). Overlay of line profiles through the centroid of the cell contact from images taken between 10 and 15 min of activation (C), or 30–35 min (D) of activation. (E) To compare variation in multiple cells (n = 10) between the two timeframes, the standard deviation of mean intensity over 5 min for each pixel along the measured line was calculated for each cell. The mean standard deviation for each cell was calculated and plotted for the 10–15 min and 30–35 min timeframes. (F) The standard deviation of pixel intensity change over a stationary line was calculated as in (E) for at least 10 cells from 2 experiments following DMSO or sequential jasplakinolide and latrunculin A treatment after 10 min of activation (*** p<0.0001). (TIF) [file pbio.1001151.s009.tif]

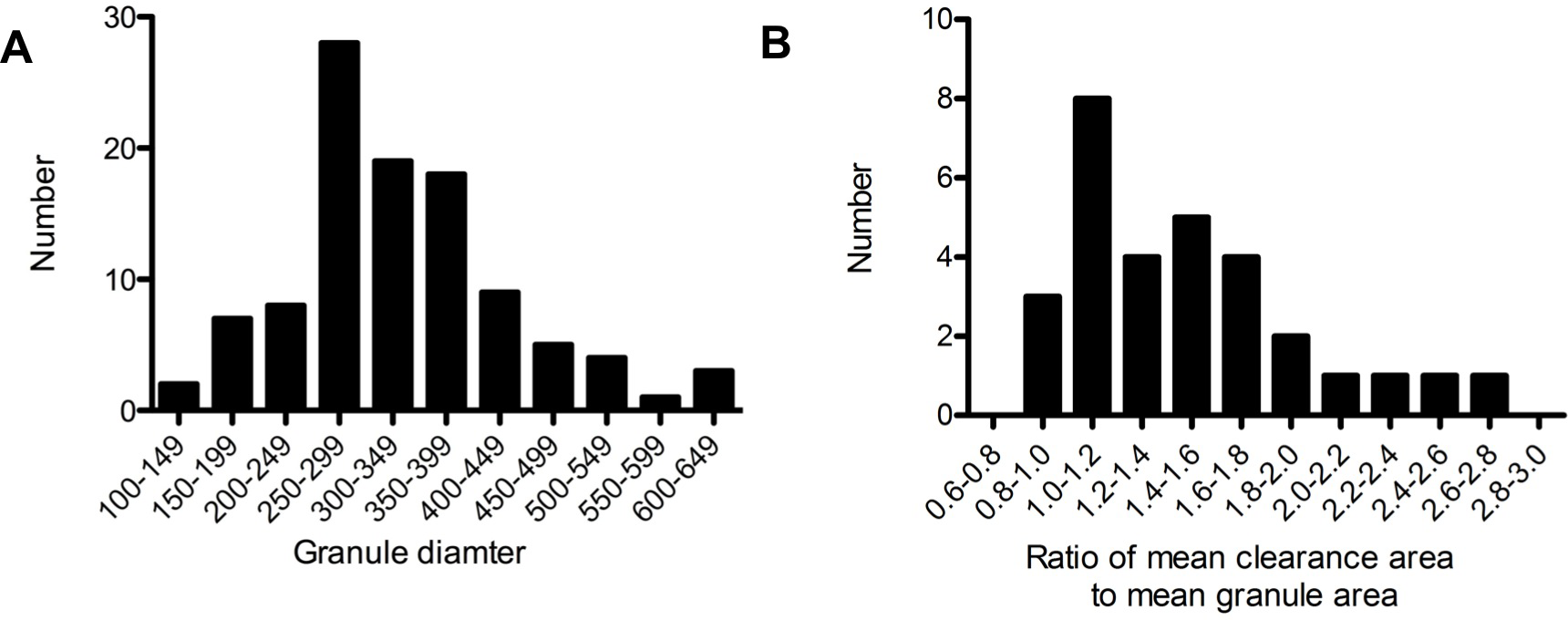

Supplement: Figure S10 — Diameters of granules imaged by STED microscopy and their relation to clearance area. (A) NK-92 cells were activated on glass, fixed, and stained for perforin. 104 granules were measured. (B) The mean clearance area for each cell (defined as any area large enough to accommodate a 250 nm in diameter granule) was divided by the mean granule equatorial area derived from (A) and plotted according to interval. The mean granule diameter of 333 nm corresponds to a mean equatorial area of 0.0871 µm2. (TIF) [file pbio.1001151.s010.tif]

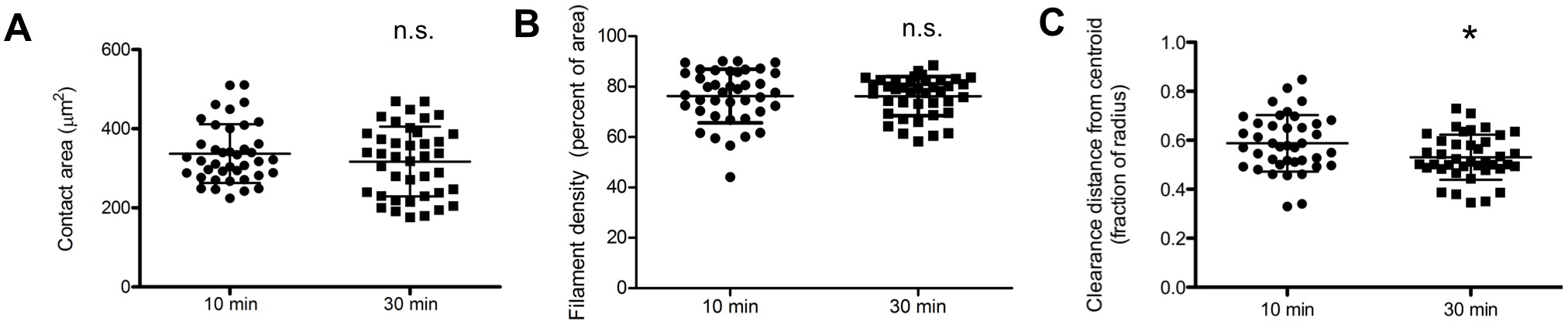

Supplement: Figure S11 — Branched networks at the activated IS. (A) High magnification image of filaments at the activated synapse using platinum replica electron microscopy. (B) Image from (A) with pseudocolored region indicating examples of branching filaments. Scale bar = 100 nm. (TIF) [file pbio.1001151.s011.tif]

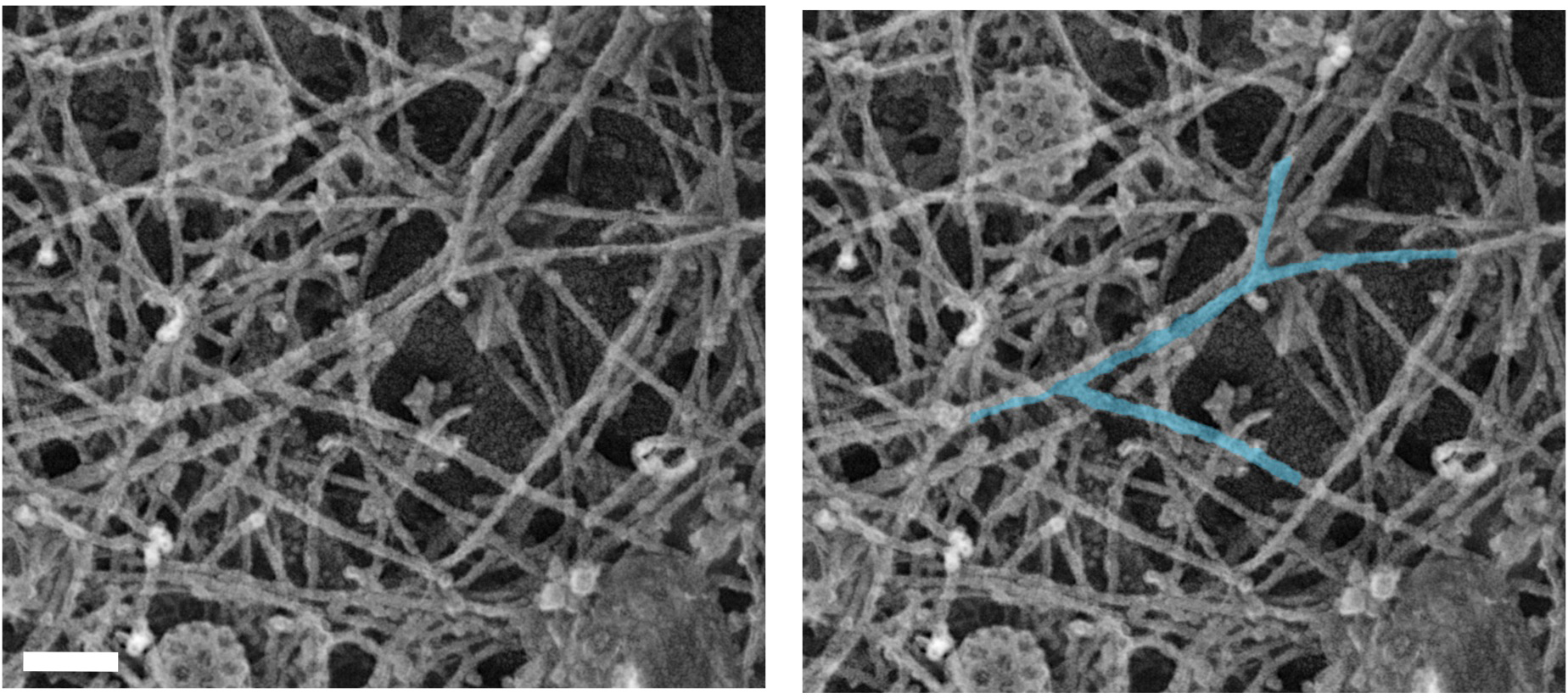

Supplement: Figure S12 — Additional analyses of cells imaged by platinum replica electron microscopy. (A–C) Comparative measurements of the synapse include: contact area (A); filament density (B); and distance from the cell centroid of individual clearances that would be greater than or equal in size to the equatorial area of a 250 nm granule (C) (* p<0.05, unpaired t test). (TIF) [file pbio.1001151.s012.tif]

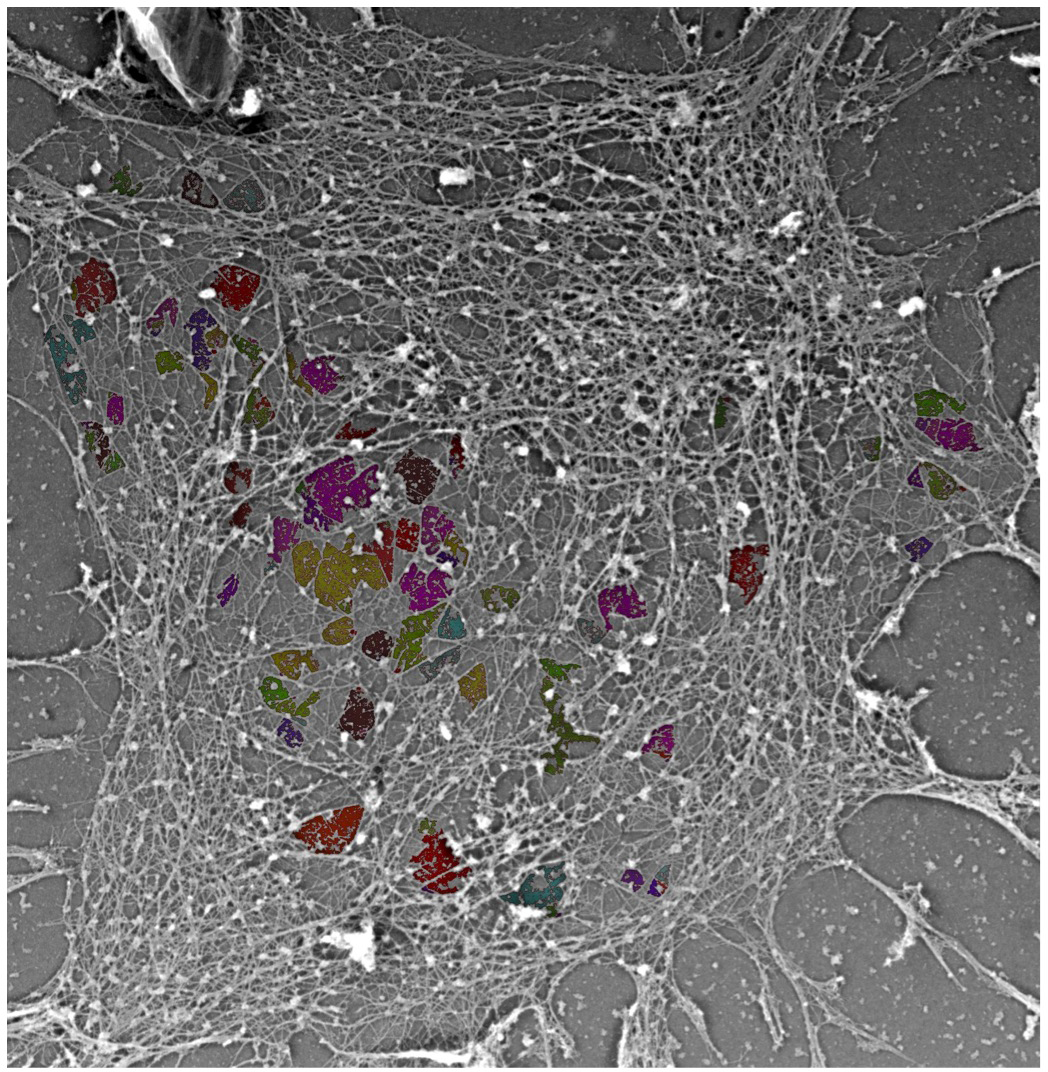

Supplement: Figure S13 — Algorithm-based identification of clearances in the F-actin network. Colored regions indicate appropriately sized clearances that were identified. (TIF) [file pbio.1001151.s013.tif]
